# Phenotyping and Genotype × Environment Interaction of Resistance to Leaffolder, *Cnaphalocrocis medinalis* Guenee (Lepidoptera: Pyralidae) in Rice

**DOI:** 10.3389/fpls.2019.00049

**Published:** 2019-02-18

**Authors:** Padmavathi Chintalapati, Divya Balakrishnan, Tripura Venkata Venu Gopal Nammi, Sumalatha Javvaji, Sampath Kumar Muthusamy, Subba Rao Lella Venkata, Sarla Neelamraju, Gururaj Katti

**Affiliations:** ^1^Indian Council of Agricultural Research, Indian Institute of Rice Research, Hyderabad, India; ^2^Entomology Division, Professor Jayashankar Telangana State Agricultural University, Hyderabad, India; ^3^National Bureau of Agricultural Insect Resources, Bengaluru, India

**Keywords:** leaffolder, resistance, stability, phenotyping traits, GGE, cluster, AMMI

## Abstract

Rice leaffolder, *Cnaphalocrocis medinalis* is one of the key foliage feeding insects of great concern throughout Asia as it results in significant yield losses. High visibility of damage is triggering farmers to apply toxic pesticides for its management. Therefore, it is vital to identify new stable sources of resistance for leaffolder. Phenotyping of 160 recombinant inbred lines (RILs) of a cross between a resistant parent, W1263 and a susceptible parent, TN1 using a rapid field screening method for three seasons resulted in identification of nine RILs as stable sources of resistance to rice leaffolder. Phenotypic frequency distributions were found continuous indicating that the resistance is a quantitative trait governed by polygenes. Phenotypic data for three seasons were analyzed using Genotype and Genotype × Environment Interaction (GGE) analysis for identification of stable resistant lines. Additive main effect and multiplicative interaction (AMMI) analysis showed that 86.41% of the total sum of square of damaged leaf area was attributed to genotype (GEN) effect; 0.48% to environment (ENV) effects and 5.68% to genotype by environment (G × E) interaction effects. Damage area, damage score and leaf length showed very high broad-sense heritability across three environments. However, leaf width had low heritability indicating higher environment influence. Phylogenetic analysis grouped these 160 RILs and parents into five clusters based on resistant reaction. AMMI and GGE biplot analysis revealed that stable genotypes G8 (MP114) and G3 (MP108) with lower damage area and damage score can be utilized in developing cultivars with leaffolder resistance.

## Introduction

Rice (*Oryza sativa* L.) is a predominant food crop of the world and staple food for about 2 billion people in the developing countries. Rice is grown in an area of 163.3 million hectares in the world with production of 749.7 million tonnes (Food and Agriculture Organisation, 2016). India is the world’s second largest producer of rice cultivated in an area of 43.9 million hectares with an annual production of 104.3 million tonnes (Directorate of Economics and Statistics, Govt. of India, 2016). Rice is the principal food crop in southern and eastern part of India and is very important in terms of national food security. Biotic stresses caused by insect pests, diseases and weeds are the major constraints in rice production resulting in 25–30% yield losses (Salim et al., 2001).

Rice leaffolder, *Cnaphalocrocis medinalis* Guénee (Lepidoptera: Pyralidae) is a notable leaf feeding insect in all the major rice growing regions in Asia. Many Asian countries like China, India, Japan, Korea, Malaysia, Sri Lanka, and Vietnam reported frequent outbreaks of this pest and yield losses (Khan et al., 1988; Luo, 2010; Li et al., 2012; Sun et al., 2013). Leaffolder infestation occurs right through the nursery stage to harvest stage, but incidences are high in the reproductive and ripening stages (Litsinger et al., 2006). Larva stitches the leaf edges and folds the leaves longitudinally. It feeds by scraping the green mesophyll tissue staying inside the folded leaves resulting in linear membranous damage. Due to this feeding, general vigor and photosynthetic efficiency of infested rice plant becomes drastically reduced resulting in poor grain filling causing significant yield loss. Initial first and early second-instar larvae are found in groups and feeds on central furled leaf. Later, larva becomes solitary and folds the leaves for feeding. Each larva can destroy several leaves by its feeding during the development passing through five instars. Due to numerous folded and damaged leaves, heavily infested fields appear parched (Padmavathi et al., 2013).

Padmavathi et al. (2013) quantified the yield losses caused by rice leaffolder and found that more than three larvae per hill at maximum tillering stage resulted in 20% unfilled grains, 57% reduction in PS II activity and 23% reduction in relative water content in comparison with the undamaged plants. They also reported that flag leaf damage of above 25% at flowering stage caused more than 50% unfilled grains indicating direct effect of yield reduction in rice. At present, farmers are dependent on chemical control as feasible method to check leaffolder infestation during the crop growth period. Though, host plant resistance plays a major role in integrated pest management, rice cultivars resistant to leaffolder are not available. Hence, farmers mainly rely on toxic pesticides leading to higher cost of cultivation and pollution hazards.

Growing resistant variety plays a major role in the management of insects especially in low input farming situations of India and South Asia. It is also highly compatible with other methods of pest management. Screening for insect resistance under natural field conditions is a long term process. At the same time, it is difficult to identify reliable and stable sources of resistance due to variation in insect populations in space and time. In addition to season specific adaptation, stability of expression of resistance to insects is also important in attaining sustainable crop yields across a wide range of environments. As the influence of environment is comparatively higher in biotic stress involving different organisms, it is essential to study the G × E interaction to quantify the stable genotypes and traits for biotic stress tolerance across different environments. Additive Main Effects and Multiplicative Interaction (AMMI) and Genotype and Genotype × Environment Interaction (GGE) biplot models are excellent tools to study G and E interactions. The AMMI model proposed by Gauch (2006) collectively considers environment (E), genotype (G), and their interaction with each other (G × E) as individual parameters for the evaluation purpose. The GGE biplot developed by Yan and Kang (2003) evaluates the interaction by considering the genotype (G) and genotype’s environmental interaction (GE). However, in recent past, AMMI method is used frequently as it couples classical additive main effects to G × E interaction values which help to ease the selection procedure and are more effective in choosing stable genotypes (Crossa et al., 1990; Ariyo and Ayo-Vaughan, 2000; Yan and Hunt, 2001; Rodrigues et al., 2014; Lado et al., 2016; Parihar et al., 2017).

As per the AMMI method, the environment (E), genotype (G) and their interaction with each other (G × E) were considered as individual parameters. This method stands as most effective for choosing genotypes in agricultural and research purpose. AMMI method also obviates the structural variations among the genotypes in environments which gives more data precision. However, the GGE parameter gives more comprehensive information in mega environments for the selection of genotypes. The information on stability and adaptability is worth considering for selection of location or seasonal specific genotypes as well as for general adaptation (Gauch, 2006). Keeping these in view, the present study was aimed to phenotype a mapping population of TN1/W1263 recombinant inbred lines (RILs) using a rapid and reliable field screening method and to identify selective phenotype traits and stable RILs resistant to rice leaffolder for utilization in crop improvement programs.

## Materials and Methods

### Plant Material

A mapping population of 160 RILs in F_10_ generation derived from a cross between two indica rice genotypes *viz*., Taichung Native 1 (TN1), a semi-dwarf susceptible variety and W1263, a resistant cultivar for rice leaffolder, was used in the study. TN1 is the world’s first semi dwarf rice variety developed from a cross between Dee-Geo-Woogen and Tsai-yuan-chunj (a Taiwanese local variety) by the Taichung District Agricultural Improvement Station in 1949. W1263 is a gall midge resistant cultivar possessing Gm1 gene developed from a cross between Eswarkorra and MTU15 by Rice Research Station, Warangal, India. Initial screening of these parents revealed W1263 as resistant to rice leaffolder. This RIL population between W1263 and TN1, developed by Dr. J. S. Bentur and his team (Biradar et al., 2004; Sundaram, 2007), available with Entomology section, ICAR-Indian Institute of Rice Research (IIRR), Hyderabad was used for phenotyping for leaffolder resistance in the present study.

### Insect Culture

Leaffolder culture was maintained on TN1 plants in the green house at 25 ± 5°C temperature and 60 ± 10% relative humidity. Adult moths were paired (10 pairs) and released for oviposition on 25–30 days old TN1 plants enclosed in a cylindrical mylar cage (14 cm diameter and 50 cm height). Adult moths were changed every day on to fresh TN1 plant. Eggs were allowed to hatch and 25–30 first instars were shifted on to a fresh TN1 plant of same age for further development. From this stock, third instar larvae (10 day old larvae) were used for phenotyping studies.

### Phenotyping of Parents and RIL Population for Resistance to Rice Leaffolder

**FIGURE 1:**
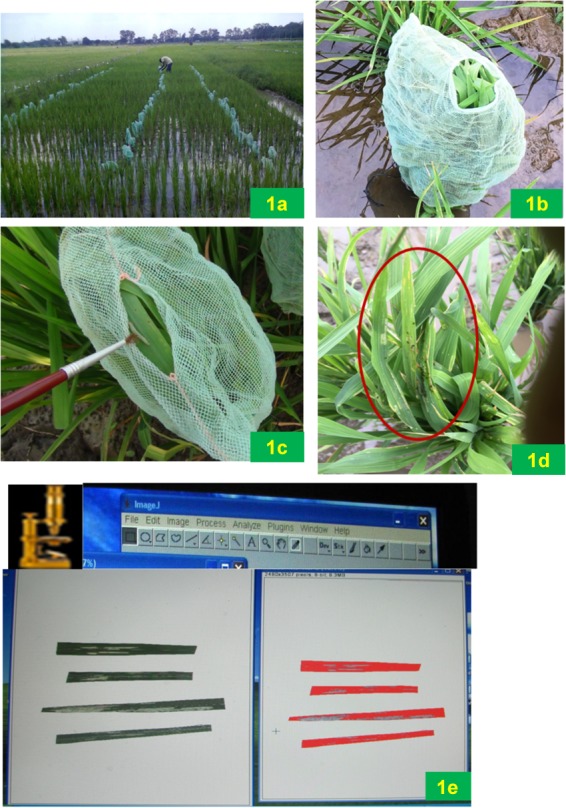
Field screening method of phenotyping for resistance to rice leaffolder; **(a)** Field lay out; **(b)** Covering of plant with a net; **(c)** Releasing third instar larva; **(d)** Damaged leaves with larva after 48 h; **(e)** Damaged area assessment using imageJ software.

Field experiments were conducted at research farm, ICAR-IIRR (17°19′N and 78°29′E), Hyderabad, Telangana State, India. This region has predominantly semi- arid type of climate with temperature range of 22–42°C and an average annual rainfall of 896 mm. The reaction of 160 F_10_ RILs along with their parents was assessed by a rapid screening method (Padmavathi et al., 2017) during three seasons viz., wet season 2013 (E1), dry season 2013–2014 (E2) and wet season 2014 (E3). These 160 RILs were grown in three blocks in a randomized manner. In each block every RIL was grown in a row of 45 hills and both the parents (TN1 and W1263) were repeated after 20 rows of test lines. RILs along with parents were grown in the field at 30 cm inter-row spacing and 20 cm intra-row spacing. All the agronomic practices recommended for raising the crop were followed. In each RIL, three plants were selected at random and screened in every block. Each time, both the parents were also screened along with the RILs. In this phenotyping method, leaves of each RIL was covered with a nylon mesh bag and tied at the bottom. A single third instar larva was released on to the leaves from the top of the bag by opening the thread and allowed to feed for 48 h (Figure 1). After 48 h, larva was collected and the number of damaged leaves were counted, collected and preserved in a book for the measurement of damaged leaf area. These damaged leaves were scanned using Cannon MF 4320–4350 scanner at color mode with 300 dpi image quality. Leaf area damaged was measured using imageJ software^1^. It took about 2 weeks for complete screening of 160 RILs along with parents. The damaged area (DA) recorded was converted to adjusted damaged area rating (ADAR) using the following formula and the percentages were converted to 0–9 scale representing the damage score (DS).

$$\mathrm{Adjusted damaged area rating}(\mathrm{ADAR})=\frac{\mathrm{Damaged area}({\mathrm{mm}}^{2})\mathrm{in test entry}}{\mathrm{Damaged area}({\mathrm{mm}}^{2})\mathrm{in susceptible check}}\times 100$$

**Table:** 

Scale	ADAR
0	no damage
1	1–10%
3	11–30%
5	31–50%
7	51–75%
9	more than 75%

RILs with mean scale score of 0–3 were considered as resistant, 3.1–5.0 as moderately resistant and 5.1–9 as susceptible.

### Characterization of Parents and RILs for Leaf Morphological Traits

Leaf morphological traits like leaf length (LL), leaf width (LW), and trichome density serve as defense factors leading to resistance, particularly, when the insect comes in contact with them and affects its growth and development after feeding. LL and LW were found to affect the folding and feeding behavior of leaffolder larva while trichome density affects the oviposition and survival of first instars. These morphological traits may have positive or negative influence on the pest and sometimes on its natural enemies also. Parents and RILs were also characterized for various morphological traits like LL and LW in all the three seasons. In each season, observations on LL and LW were recorded from five leaves selected randomly from five hills in each RIL at 50 days after planting. Length of the leaf was measured with a standard scale from leaf tip to the point at which lamina is attached to the petiole. LW was measured at the widest part of leaf lamina from one edge to other edge. The number of trichomes on both, adaxial and abaxial leaf surfaces was also recorded by taking 5 cm leaf portion from the middle of the second leaf as per the procedure described by Maiti and Gibson (1983). In this method, three plants from each RIL were selected at random and leaf samples were collected. Leaf samples were cut into 5 cm leaf bits in the middle of the leaf, dipped in 1:2 alcohol, acetic acid solution and left overnight for the clearance of chlorophyll content. These discolored leaf bits were stored in vials with 90% lactic acid. Leaf bits were mounted on a glass slide with lactic acid and observed under compound microscope (OLYMPUS BX50). Trichomes on abaxial and adaxial surfaces of each leaf were counted and expressed as numbers per microscopic field.

### Statistical Analysis

Descriptive statistics such as mean, standard error (SE), range, coefficient of variation (CV%), Analysis of Variance (ANOVA) and heritability for each leaf morphological trait, and correlations among pairs of traits were calculated using the Plant Breeding Tools (PB Tools, 2014) software. ANOVA was used to compare the variation in resistance among the testing environments (E) and among the entries (RILs) within population and G × E interactions of the 160 RILs and to compare the resistance reaction between the entries and parental checks evaluated in different testing environments. Significantly different lines compared to parents were identified using significant pairwise mean comparison method. Mean phenotypic data was subjected to cluster analysis using DARwin software version 5.0 (Perrier and Jacquemoud-Collet, 2006)^2^. Unweighted Pair Group Method with Arithmetic Mean (UPGMA) was used to generate a dendrogram based on calculated phenotypic dissimilarity on euclediean distances for the 160 RILs.

### AMMI and GGE Analysis

The AMMI and GGE models were applied for stability analysis of 160 RILs, with two parents (Genotype = G) and three testing seasons (Environments = E) and their genotype by environment (G × E) interactions. AMMI and GGE statistical models and computational methods were used in the current study as described in Mukherjee et al. (2013), Gauch (2013), and Balakrishnan et al. (2016). The ANOVA were employed to partition the variation into RILs (G) main effects and environments (E) main effects and genotype by environment (G × E). AMMI and GGE biplots were used to partition the G × E interaction into several principal components (Supplementary Table S1). The ANOVA, AMMI biplot, and GGE analysis were carried out by software program Plant Breeding Tools Version 1.4 (PB Tools, 2014) developed by International Rice Research Institute (IRRI), Philippines.

## Results

### Phenotypic Variation in Parents and RIL Population

**FIGURE 2:**
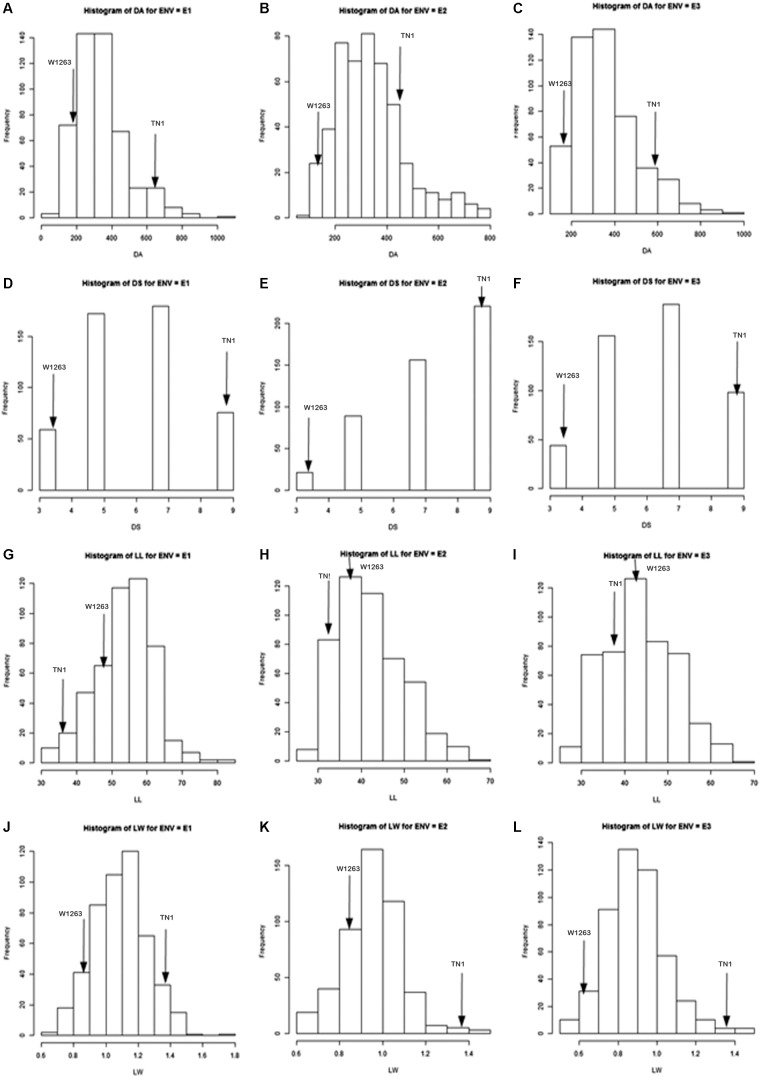
Frequency distribution of leaffolder resistance and leaf related traits in a population derived from TN1x W1263; E1 = Wet season 2013; E2 = Dry season 2013–2014; E3 = Wet season 2014; DA = Damaged area; DS = Damage score; LL = Leaf length; LW = Leaf width. **(A)** DA during wet season 2013; **(B)** DA during dry season 2013–2014; **(C)** DA during wet season 2014; **(D)** DS during wet season 2013; **(E)** DS during dry season 2013–2014; **(F)** DS during wet season 2014; **(G)** LL during wet season 2013; **(H)** LL during dry season 2013–2014; **(I)** LL during wet season 2014; **(J)** LW during wet season 2013; **(K)** LW during dry season 2013–2014; **(L)** LW during wet season 2014.

The mean phenotypic variation for damaged area and damage score showed a continuous normal distribution in all the three seasons (Figure 2). During wet season 2013, damage area of only 195.04 mm^2^ was recorded in the resistant parent, W1263 while it was 606.05 mm^2^ in TN1, the susceptible parent (Figure 2A). Damaged area in RILs varied from 71.08 to 1068.70 mm^2^. The leaffolder damage was comparatively less during dry season 2013–2014 with damaged area of 142.86 mm^2^ in W1263 and 440.14 mm^2^ in TN1 (Figure 2B). Damaged area in RILs varied from 87.35 to 779.73 mm^2^ (Table 1). Similarly, during wet season 2014, damaged area ranged between 107.84 and 945.90 mm^2^ with 171.75 mm^2^ in W1263 and 605.21 mm^2^ in TN1 (Figure 2C). The mean damage score varied from 3.0 to 9.0 in different RILs in all the three seasons with a damage score of 3.0 in W1263, the resistant parent, and 9.0 in TN1, the susceptible parent. A total of nine RILs were found resistant to rice leaffolder with a damage score of 3.0 while 42 RILs were found moderately resistant with a damage score of 3.7–5.0 (Figure 2D–F).

**Table 1:** The mean performance and descriptive statistics of traits under the study across the environments.

Variable	ENV	Min	Max	Mean	Range	Variance	Std Dev	CV	Skewness	Kurtosis	PCV	GCV	h^2^	GA	S.E.	CV (%)
DA	E1	71.08	1068.70	338.32	997.62	21350.81	146.12	43.19	1.12	1.80	43.27	40.80	88.90	1.73	28.16	14.41
DA	E2	87.35	779.73	335.99	692.38	18775.50	137.02	40.78	0.88	0.75	40.83	38.58	89.25	1.74	25.97	13.39
DA	E3	107.84	945.90	358.34	838.06	21171.76	145.51	40.60	0.93	0.88	40.69	38.16	87.95	1.70	29.22	14.12
DS	E1	3.00	9.00	6.12	6.00	3.21	1.79	29.30	−0.03	−0.76	29.34	26.31	80.38	1.48	0.46	13.00
DS	E2	3.00	9.00	7.37	6.00	3.11	1.76	23.93	−0.75	−0.42	23.93	21.41	80.03	1.47	0.45	10.70
DS	E3	3.00	9.00	6.40	6.00	3.18	1.78	27.89	−0.14	−0.76	27.93	25.85	85.61	1.63	0.39	10.60
LL	E1	31.00	82.00	54.26	51.00	70.00	8.37	15.42	−0.16	0.26	15.44	13.66	78.26	1.43	2.26	7.20
LL	E2	27.00	66.00	42.55	39.00	58.58	7.65	17.99	0.61	−0.08	18.02	16.83	87.30	1.68	1.58	6.42
LL	E3	28.00	66.00	44.04	38.00	65.47	8.09	18.37	0.26	−0.53	18.40	17.26	87.98	1.70	1.62	6.38
LW	E1	0.60	1.80	1.14	1.20	0.03	0.17	14.74	0.10	0.17	14.77	10.64	51.87	0.77	0.07	10.25
LW	E2	0.60	1.50	1.00	0.90	0.02	0.14	13.84	0.02	0.94	13.83	10.43	56.84	0.88	0.05	9.09
LW	E3	0.50	1.50	0.94	1.00	0.02	0.16	16.64	0.55	1.04	16.67	14.02	70.68	1.22	0.05	9.03

*DA, damaged area; DS, damage score; LL, leaf length; LW, leaf width; E1 = wet season 2013; E2 = dry season 2013–2014; E3 = wet season 2014.*

### Characterization of Leaf Morphological Traits in Parents and RIL Population

**FIGURE 3:**
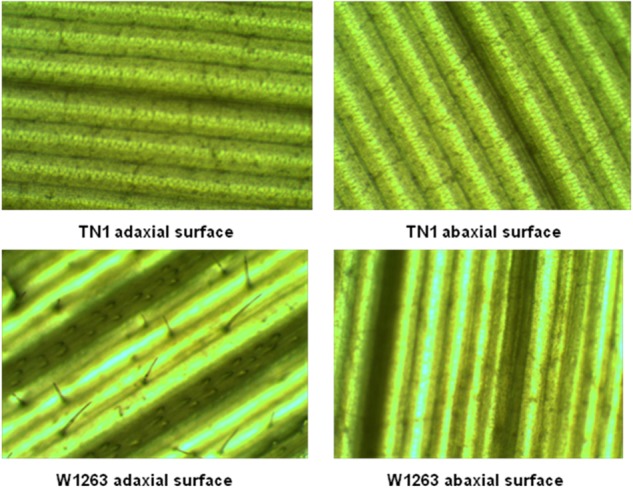
Trichomes on leaf surfaces of TN1 (susceptible parent) and W 1263 (resistant parent).

There were significant differences among RILs for leaf morphological traits like LL, LW, and trichome density. LL and width traits of RIL population also exhibited a continuous normal distribution in all the three seasons. The mean LL varied from 31.0 to 82.0 cm in different RILs with 48 cm in W1263 and 37 cm in TN1 whereas LW ranged between 0.6 and 1.8 cm in different RILs with 0.8 cm in W1263 and 1.5 cm in TN1 during wet season 2013 (Figure 2G,J). Similarly, mean LL varied from 28.0 to 66.0 cm and LW from 0.5 to 1.5 cm in different RILs during wet season 2014 (Figure 2H,K). LL of 44.0 cm, 36.7 cm, and LW of 0.7 cm, 1.4 cm was recorded in W1263 and TN1, respectively. During dry season 2013–2014, LL ranged between 27.0 and 66.0 cm while LW ranged between 0.6 and 1.5 cm in various RILs (Table 1). Trichome density varied from 0 to 396 per objective area on adaxial surface of the leaf and ranged between 0 and 142 on abaxial surface in different RILs (Table 2). Trichomes were completely absent on both adaxial and abaxial surfaces in the susceptible parent, TN1 (Figure 3), while resistant parent, W1263 showed more numbers on adaxial surface (314/objective area) as compared to abaxial surface (45/objective area). Correlation studies revealed a negative relationship between trichome density and leaffolder damage but it was not-significant (*r* = −0.0391; *P* = 0.6463).

**Table 2:** Trichome density (Mean number/microscopic field) on adaxial and abaxial leaf surfaces in different RILs of mapping population of TN1/W1263.

RIL No	Trichome density		RIL No	Trichome density		RIL No	Trichome density		RIL No	Trichome density	
	Adaxial	Abaxial		Adaxial	Abaxial		Adaxial	Abaxial		Adaxial	Abaxial
2	215	0	115	29	0	226	390	44	342	77	0
4	109	6	116	142	7	227	247	38	348	230	21
7	249	0	117	283	0	228	252	15	353	259	12
8	201	0	120	161	0	230	329	122	355	326	0
9	186	0	121	121	0	231	333	39	357	166	0
10	115	0	122	166	6	232	202	0	425	256	0
11	145	0	123	138	12	233	275	0	434	229	64
12	218	0	124	171	0	234	348	37	435	175	0
14	314	0	125	179	0	235	218	66	436	288	54
15	168	5	126	172	3	236	206	43	440	94	0
16	245	3	127	253	0	237	254	26	442	186	54
17	270	0	131	374	104	241	121	26	443	221	5
18	248	0	132	235	75	243	178	10	457	83	0
19	216	0	133	314	0	244	340	0	459	195	0
20	173	0	135	115	7	245	235	0	460	176	16
21	298	0	138	283	74	246	316	12	520	77	10
22	311	91	139	244	39	247	185	3	524	160	0
23	300	0	142	290	0	248	228	6	528	290	0
27	305	27	143	337	53	249	293	0	530	146	0
28	167	0	144	320	0	301	230	42	531	170	0
31	71	0	145	378	97	307	1	0	533	23	0
32	331	112	146	235	0	312	395	76	536	3	0
35	288	0	148	323	79	313	349	8	537	104	0
37	192	0	149	248	51	314	229	23	539	43	4
40	160	9	206	167	1	316	333	4	540	219	19
42	171	5	209	321	58	317	146	0	541	222	0
44	200	7	212	119	15	319	71	0	542	144	1
45	299	0	215	314	0	320	190	0	544	226	10
46	216	0	216	161	22	323	229	3	547	123	1
107	228	56	217	146	3	327	323	4	549	281	36
108	167	0	220	396	32	331	394	0	553	133	0
110	258	0	221	327	142	337	252	0	556	96	0
111	0	0	222	229	33	338	67	0	558	376	0
112	198	0	223	175	16	339	173	15	TN1	0	0
114	293	10	224	234	73	340	208	0	W1263	314	45
		Adaxial surface		Abaxial surface							
Min		0		0							
Max		396		194							
LSD (0.05)		2.10		1.97							

*All values were converted to square root transformed values (x + 0.5) before analysis. DA, damaged area; DS, damage score; LL, leaf length; LW, leaf width.*

### Phylogenetic Analysis of the Population

**FIGURE 4:**
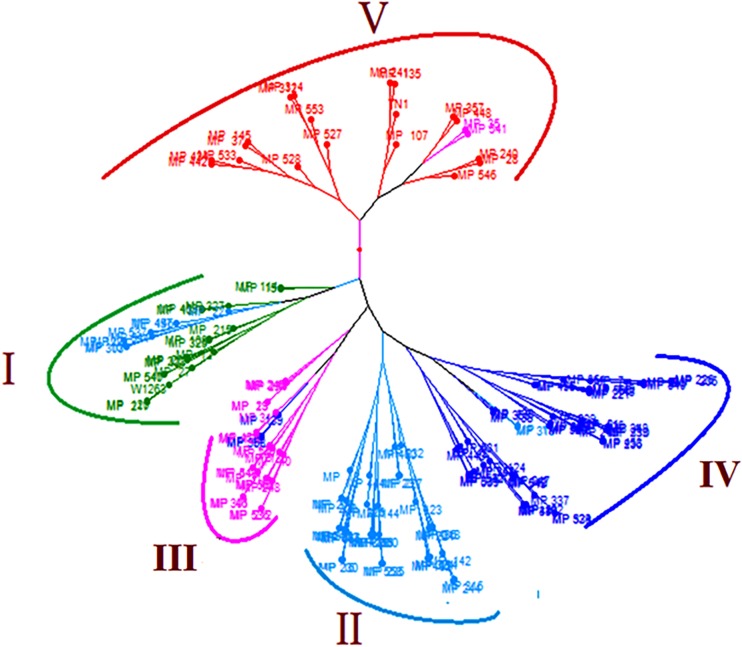
Cluster diagram illustrating the genetic relationship among the 160 RILs and parents. Cluster I (DA = 112.18–195.81 mm^2^; DS = 3.0–4.6), Cluster II (DA = 200.91–298.40 mm^2^; DS = 4.3–6.3), Cluster III (DA = 301.59–397.33 mm^2^; DS = 6.1–7.9), Cluster IV (DA = 405.97–497.93 mm^2^; DS = 7.4–8.6), Cluster V (DA = 508.01–780.51 mm^2^; DS = 9.0).

The clusters based on mean phenotypic data showed very clear distinguished grouping of RILs according to their resistance reaction and corresponding scores. The tree showed five large clusters (I–V) in which RILs and parents were grouped based on their resistance reaction (Figure 4). RILs in cluster I belonged to resistant and moderately resistant class with a DA of <200 mm^2^, DS of 3.0–4.6. Cluster II also had moderately resistant RILs with DA of 200.91–298.40 mm^2^, DS of 4.3–6.3. Susceptible RILs formed three clusters, Cluster III with DA of 301.59–397.33 mm^2^, DS of 6.1–7.9; cluster IV with DA of 405.97–497.93 mm^2^, DS of 7.4–8.6 and cluster V with DA of 508.01–780.51 mm^2^, DS of 9.0. Cluster V had RILs with DA of more than the susceptible check. Few admixtures belonging to the adjacent clusters were found in all the clusters except in the cluster II.

### Trait Performance of the Parents and Population

The population was evaluated for its resistance to leaffolder in each individual RIL raised in RCBD (Randomized complete Block design) for three seasons (wet season 2013, 2014, and dry season 2013–2014) according to rapid screening method along with parents. ANOVA was used to compare the variation in resistance among the environments (E) and among the RILs (G) within population and G × E interactions of the 160 RILs and to compare the resistance reaction between the RILs and parental checks evaluated in different testing environments. ANOVA showed significant genotype by environment interactions for these traits. Of 160 tested RILs, 130 were found to be significantly resistant in terms of damage area (81.25% of tested RILs) and 93 showed resistance in terms of damage score over TN1. Only 98 RILs (58.12% of tested RILs) were significantly susceptible to resistant parent W1263. Among the tested RILs, 20 (12.5% of tested RILs) were found to be positively significant for LL and 136 were negatively significant for LW of TN1. These results indicated that the majority of the population showed resistance to the rice leaffolder. The overall mean and range of traits measured to assess the resistance reaction during wet seasons of 2013, 2014 and dry season of 2013–2014 are presented in Table 1. A wide variation was observed for damaged area, damage score, LL and LW in this set of population (Table 1). Among the traits, damage area and LL were observed to have wide variability and showed more environment influence. Highest mean for DA was observed in E3 whereas highest mean damage score was observed in E2. Skewness and Kurtosis were measured to describe the nature of distribution (Table 1). All four traits showed platykurtic distribution (kurtosis value < 3) across three environments. Heritability estimates in a broad-sense for DA, DS, LL, and LW were observed and all the traits except LW showed high level of heritability. Strong highly significant correlation existed between mean DA and DS, and significant correlation between mean LL and LW. However, correlation was positive between damage area and damage score with LW and negative with LL (Table 3).

**Table 3:** The correlation matrices of mean performance of leaffolder resistance and leaf related traits across three environments.

		E1				E2				E3			
		DA	DS	LL	LW	DA	DS	LL	LW	DA	DS	LL	LW
E1	DA	1.00											
	DS	0.90^∗∗∗^	1.00										
	LL	0.13	0.16	1.00									
	LW	0.41^∗∗^	0.47^∗∗^	0.04	1.00								
E2	DA	0.94^∗∗∗^	0.85^∗∗∗^	0.15	0.39	1.00							
	DS	0.76^∗∗∗^	0.80^∗∗∗^	0.10	0.54^∗∗∗^	0.82^∗∗∗^	1.00						
	LL	−0.07	−0.01	0.36^∗∗^	0.01	−0.05	−0.01	1.00					
	LW	0.49^∗∗∗^	0.48^∗∗∗^	−0.06	0.55^∗∗∗^	0.47^∗∗∗^	0.51^∗∗∗^	−0.03	1.00				
E3	DA	0.89^∗∗∗^	0.81^∗∗∗^	0.13	0.41^∗∗^	0.90^∗∗∗^	0.72^∗∗∗^	−0.01	0.49	1.00			
	DS	0.81^∗∗∗^	0.82^∗∗∗^	0.17	0.50^∗∗^	0.84^∗∗∗^	0.80^∗∗∗^	0.01	0.49^∗∗∗^	0.92^∗∗∗^	1.00		
	LL	−0.17	−0.18	0.16	0.01	−0.17	−0.18	0.32^∗∗^	−0.11	−0.14	−0.14	1.00	
	LW	0.42^∗∗^	0.44^∗∗^	0.04	0.64^∗∗∗^	0.40	0.47^∗∗^	0.02	0.56^∗∗∗^	0.43^∗∗^	0.45^∗∗^	0.11	1.00

*Significance levels: ^∗^P < 0.05, ^∗∗^P < 0.01, and ^∗∗∗^P < 0.001. DA, damaged area; DS, damage score; LL, leaf length, LW, leaf width; E1 = wet season 2013; E2 = dry season 2013–2014; E3 = wet season 2014.*

### AMMI Analysis of Variance

**FIGURE 5:**
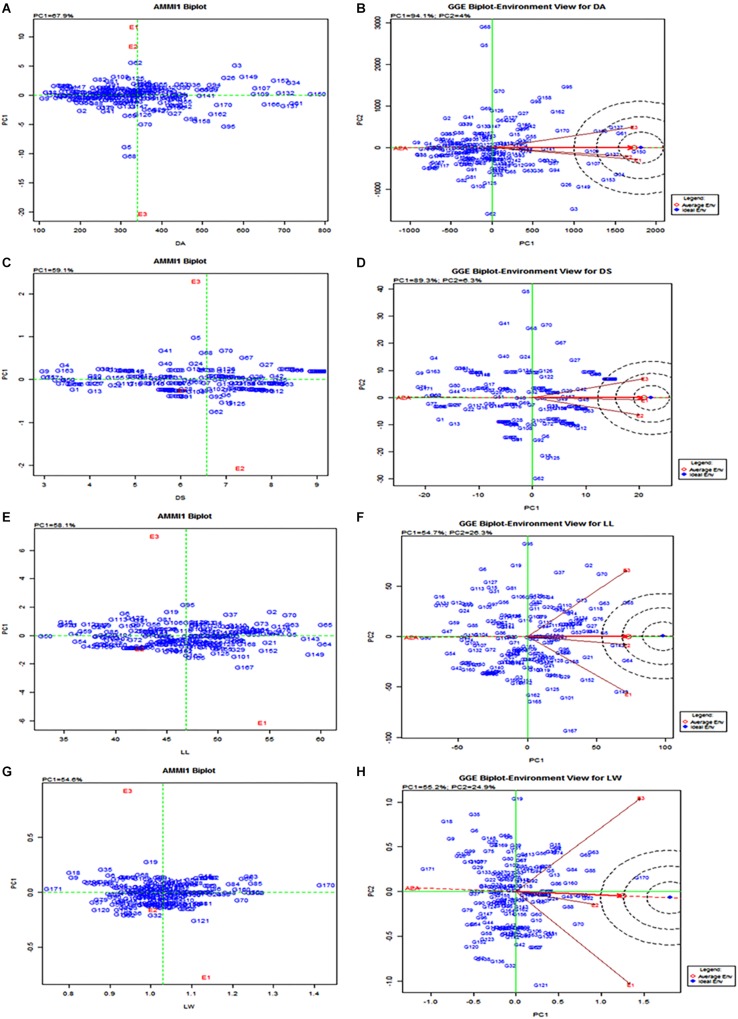
AMMI and GGE biplot for the primary component of interaction (PC1) and mean or main effect of RILs in different seasons. E1 = Wet season 2013; E2 = Dry season 2013-2014; E3 = Wet season 2014; DA = Damage area; DS = Damage score; LL = Leaf length; LW = Leaf width. **(A)** AMMI biplot for DA; **(B)** GGE biplot for DA; **(C)** AMMI biplot for DS; **(D)** GGE biplot for DS; **(E)** AMMI biplot for LL; **(F)** GGE biplot for LL; **(G)** AMMI biplot for LW; **(H)** GGE biplot for LW.

The AMMI analysis of variance of 160 RILs tested against leaffolder resistance for three seasons (environments) showed that 86.41% of the total sum of square (SS) of damaged area was attributed to genotype (GEN) effect; 0.48% to environment (ENV) effect and 5.68% to genotype by environment (G × E) interactions effects (Table 4). Leaffolder resistance scores of the population ranged from three (3.0) to nine (9.0) with greater proportion of RILs showing more stable reaction status. The resistant check, G162 (W1263) recorded stable resistant reactions over all the test environments (Figure 5). RILs or environment appearing almost on the perpendicular lines to axis showed similar mean performance. Also, RILs or environment on the right side of the perpendicular lines through origin had higher leaffolder damage scores than those on the left side. Therefore, it is quite evident from the biplot that the RILs, G161(TN1), G2(MP107), G25(MP135), G33(MP145), G55(MP28), G59(MP37), G85(MP240), G86(MP241), and G98(MP312) showed high level of stable susceptibility. In contrast, G8(MP114), G49(MP215), G37(MP15), G51(MP217), G154(MP547), G3(MP108), G107(MP325), G13(MP120), G54(MP27) along with resistant check G162(W1263) showed lower damage score and greater stable resistance reactions to leaffolder (Figure 5); while G4(MP11) and G60(MP4) were few of the most responsive RILs for both DA and DS. G8(MP114) and G3(MP108) were most stable with lower damaged area while G141(MP528), G123(MP434), G33(MP145), G59(MP37), G125(MP436), G144(MP533), G98(MP312), G100(MP314), and G157(MP553) showed stable consistent performance with higher damaged leaf area across three environments. For LL, G62(MP42), G63(MP44), G134(MP453), G144(MP533), and G4(MP11) showed high mean performance and stability across environments, while G15 (MP122), G122(MP425), G46(MP21), and G57(MP32) were the stable lines with lower LL. For LW, G161(TN1) was the most stable genotype with highest mean value while the other parent G162(W1263) was most stable for lower LW and was on par with G8(MP114) and G17(MP124). All three environments i.e., E1 (wet season 2013), E2 (dry season 2013–2014), and E3 (wet season 2014) were far away from the origin and are differentiating environments and were on the right hand side of the origin of the main effect axis suggesting that these environments were favorable for leaffolder infestation. E1, E2, and E3 showed low PC1 scores for all the traits under study with small interactions (Table 4). Similarly, significantly larger proportion of RILs recorded low PC1 scores and showed small interactions for damage score and LW, which caused clustering together of the RILs on the biplot (Figure 5). However, higher proportion of RILs recorded significantly higher PC1 scores in case of damaged area and LL. RILs G138(MP520) and G76(MP228) recorded the lowest PC1 score of 0.21 and 1.37, respectively, for damaged area and so can be considered as the stable susceptible lines.

**Table 4:** The factor explained SS (%) was calculated comparing sum of square (SS) from AMMI ANOVA showing the percentage contribution of genotype environment and interaction effects in phenotypic expression of each trait across environments.

Source	DF	SS	DS	F	Pr (>F)	SS (%)
**Response Variable: DA**						
ENV	2	144236.1	72118.05	21.7	0.0018^∗∗∗^	0.48
REP within ENV	6	19943.05	3323.842	1.43	0.2011^∗^	0.07
GENO	163	26230602	160923.9	69.08	0.0000^∗∗∗^	86.41
ENV:GENO	325	1723469	5302.981	2.28	0.0000^∗∗∗^	5.68
Pooled Error	961	2238675	2329.526			7.37
Total	1457	30356924				
**Response Variable: DS**						
ENV	2	423.4179	211.709	154.46	0.0000^∗∗∗^	8.26
REP within ENV	6	8.2239	1.3707	2.39	0.0269^∗^	0.16
GENO	163	3659.947	22.4537	39.14	0.0000^∗∗∗^	71.41
ENV:GENO	325	482.4604	1.4845	2.59	0.0000^∗∗∗^	9.41
Pooled Error	961	551.2485	0.5736			10.76
Total	1457	5125.298				
**Response Variable: LL**						
ENV	2	40779.79	20389.89	1306.06	0.0000^∗∗∗^	30.01
REP within ENV	6	93.6706	15.6118	1.53	0.1665^∗^	0.07
GENO	163	44083.59	270.4515	26.43	0.0000^∗∗∗^	32.44
ENV:GENO	325	41100.05	126.4617	12.36	0.0000^∗∗∗^	30.24
Pooled Error	961	9835.036	10.2342			7.24
Total	1457	135892.1				
**Response Variable: LW**						
ENV	2	10.209	5.1045	553.48	0.0000^∗∗∗^	22.52
REP within ENV	6	0.0553	0.0092	0.95	0.4602^∗^	0.12
GENO	163	13.1225	0.0805	8.27	0.0000^∗∗∗^	28.95
ENV:GENO	325	12.5904	0.0387	3.98	0.0000^∗∗∗^	27.77
Pooled Error	961	9.3571	0.0097			20.64
Total	1457	45.3344				

*Significance levels: ^∗^P < 0.05, ^∗∗^P < 0.01, and ^∗∗∗^P < 0.001.*

### GGE Biplot – Environment View

Environment-vector view of the GGE biplot explained 93.9, 88.2, 51.9, and 52.9% of the total variation of the environment-centered G by E for DA, DS, LL, and LW, respectively (Figure 5). All the three environment vectors appeared to be positively correlated suggesting that the same information about the genotype could be obtained from fewer test environments. However, E1, E2, and E3 appear to be the more discriminating or informative in case of traits related to leaf area than leaffolder damage even though the location was same and the seasons were different. The three environments were observed to be representative in case of DA and DS with a smaller deviation from average environment axis though E2 was most representative being very close to average environment in case of LL and LW. E1 and E3 were most discriminative for LL and LW while E2 and E3 for DS and only E3 for DA. E1 was observed to be closest to ideal test environment for DS and hence considered to be the best for leaffolder screening but E2 appeared to be closer to ideal test environment for all the other three traits.

### What-Won-Where Biplot

The pattern of what won where plot suggested that the target environment may consist of only one mega-environment for DA and LW, since all three environments appeared in one sector while the environments grouped into two sectors in case of DS and LL (Supplementary Tables S2–S5). For example, some of the winners in this mega-environment were G2(MP107), G144(MP533), G143(MP531), G33(MP145), G128(MP442), and G86(MP241) as they consistently showed stable leaffolder susceptibility in all the different environments (Supplementary Figure S1). G8(MP 114) and G3(MP 108) showed stable resistance level by appearing in the extreme opposite direction of winners indicating a below average mean for both damage score and damage area which is preferable in resistance studies. For LW, which won where plot showed parents, G161 (TN1) with positive average mean and G162 (W1263) with negative average mean and appeared in extreme vertices on either side of axis going through origin showing the precise demarcation of RILs by GGE biplots for stability and mean levels of traits under study. On the other hand, RILs clustering toward the origin of the biplot consistently showed stable resistant status with average mean levels and were the most important set of entries from an entomologist and plant breeder’s perspective. The RILs crowded together at the origin of the biplot also showed some crossover effects (Supplementary Figure S1).

## Discussion

Rice leaffolder, *C medinalis* is one of the most important biotic constraints in rice production and its highly visible damage symptoms triggers farmers for application of pesticides at early crop growth stage leading to soil and environmental pollution (Chintalapati et al., 2016). Hence it is important to identify stable resistant lines for the development of rice cultivars resistant/tolerant to leaffolder. Earlier, researchers have made limited progress in identifying resistant sources for leaffolder due to lack of rapid, reliable and reproducible screening method to evaluate large number of germplasm lines in the field. In our previous study, we developed a rapid field screening method to evaluate a large number of genotypes to identify resistant sources against rice leaffolder (Padmavathi et al., 2017). Using this method, we identified TN1 as most susceptible and W1263 as resistant genotype. In the present study, we evaluated 160 RILs of a mapping population of TN1/W1263 and identified nine RILs, i.e., G8(MP114), G49(MP215), G37(MP15), G51(MP217), G154(MP547), G3(MP108), G107(MP325), G13(MP120), and G54(MP27) with lower damage score and greater stable resistance reactions to leaffolder along with resistant parent G162 (W1263). On the contrary, eight RILs viz., G2(MP107), G25(MP135), G33(MP145), G55(MP28), G59(MP37), G85(MP240), G86(MP241), and G98(MP312) showed high level of stable susceptibility along with susceptible parent G161(TN1) with high damage score. Earlier, Xu et al. (2010) reported that most varieties (or lines) cultured in rice production were susceptible to damage caused by *C. medinalis.* Yangjing 9538, 91SP, and TN1 were the most susceptible (Damage leaves scale of 7 and 9) and no highly resistant variety was found against *C. medinalis*. Elanchezhyan and Arumugachamy (2015) reported that none of the medium duration rice genotypes evaluated against rice leaffolder were free from leaf damage to be categorized as highly resistant with 0% leaf damage.

Among the morphological traits, five RILs, i.e., G62(MP42), G63(MP44), G134(MP453), G144(MP533), and G4(MP11) showed high mean performance and stability across environments with respect to LL and LW while G161(TN1) was most stable with highest mean value of LW and G162(W1263) as the most stable with lowest mean value of LW.

Correlation studies revealed a significant positive relation between damaged area and damage score with LW and negative association with LL. Similar observations were made by Chalapathi Rao et al. (2002), Nigam et al. (2008), and Xu et al. (2010) who reported that LL had no significant effect on leaffolder incidence but a significant positive correlation existed between leaf folder damage and LW. Similarly, Sarao et al. (2013) reported a significant positive correlation between flag LW and leaffolder infested leaves at vegetative (*r* = 0.63) and panicle initiation (*r* = 0.64) stages. Kamakshi et al. (2015) noticed that LW at 25 and 40 DAT had significant positive correlation with per cent leaf damage (*r* = 0.794 and 0.667, respectively). In the present study, a negative correlation was observed between leaffolder damaged area and damage score with trichome density, though not significant. However, Hakkalappanavar et al. (2011) reported a significant negative relationship between leaffolder infestation and trichome density (*r* = −0.40) and positive significant relationship (*r* = 0.53) with LW at midpoint.

Damage area, damage score and LL showed very high broad-sense heritability across three environments. However, LW had low heritability indicating higher environment influence. The stable RILs identified for DA, DS, and LW include G8(MP114), G49(MP215), G37(MP15), G51(MP217), G154(MP547), G3(MP108), G107(MP325), G13(MP120), and G54(MP27) and are the potential donors for resistance breeding programs to leaffolder. The cluster analysis also grouped the RILs into clear clusters of resistant and susceptible lines showing the accuracy of our scoring for resistance. The crosses between stable resistant and susceptible lines will be useful in genetic analysis of component traits and dissecting the mechanisms of leaffolder resistance. The stable RILs identified in this study will be tested under multi location trials to identify the suitable environments where RILs show specific adaptation.

The phenotypic frequency distributions observed in this study showed a continuous distribution indicating quantitative inheritance of leaffolder damage (Figure 2) suggesting that the resistance/susceptibility is under polygenic control. In the RIL population evaluated, 130 of 160 tested lines were found to be significantly resistant over susceptible parent, TN1 in terms of damaged area signifying predominance of resistant progenies over susceptible ones confirming the presence of major genetic loci governing resistance in this population. The transgressive segregants in the population outside the parental values are mainly due to combination and interaction of alleles from both the parents contributing for resistance reaction similar to observations recorded by Aruna et al. (2011) on 385 RILs against sorghum shoot fly. They identified five stable shoot fly resistant lines that are well adapted to all the eight environments tested. They reported that environment had the greatest effect (69.2%) on dead heart damage followed by G × E interactions (24.6%) and genotype (6.2%) indicating that shoot fly resistance is a highly complex character.

Gu et al. (2004) stated that to identify stable resistance, the host genotypes have to be exposed to repeat testing under different environments, either through multi-location trials during the same year or repeated testing at the same location for several years. Aruna et al. (2011), Beyene et al. (2011), Mukherjee et al. (2013), Asnakech et al. (2017) studied crop pest resistance and stability of resistance reaction in the genotypes, RILs and found that this helped in accurate prediction of the genotype-phenotype relationship and efficient selection of useful parental combinations.

Quantitative traits like pest resistance and yield traits are highly influenced by environment interaction effects. Significant genotype, environment and genotype × environment interaction were observed and similar results were reported by Beyene et al. (2011) for stem borer resistance in maize. AMMI and GGE biplot analysis clearly separated main and interaction effects and provided meaningful interpretations of the data and are highly useful in predicting stable resistant genotypes with high levels of resistance and low fluctuations over different environments (Ebdon and Gauch, 2002).

However, ANOVA showed a significant SS for genotype revealing the diverse nature of RILs with large variations in damage area and damage scores. The magnitude of G × E interaction and environment SSs were lower than that of genotype. This clearly indicated that the differences of the RILs across the environment were not substantial and the resistance is mainly due to genotype effect. Similarly, genotypic SS for DS was 71.41% and higher than that of environment and interactions (Table 4). But in case of LL and LW, SS% on genotype, environment and G × E were almost similar showing the contribution of all the three in expression of the phenotype. Clustering of RILs toward origin in case of LW was found in the AMMI biplot indicating a lower contribution of genotype in the G × E interaction. Testing of these identified stable genotypes in different locations will help in identification of suitable environments and RILs with specific adaptation. These identified stable RILs may be used in breeding programs aimed at developing leaffolder resistant cultivars.

**FIGURE 6:**
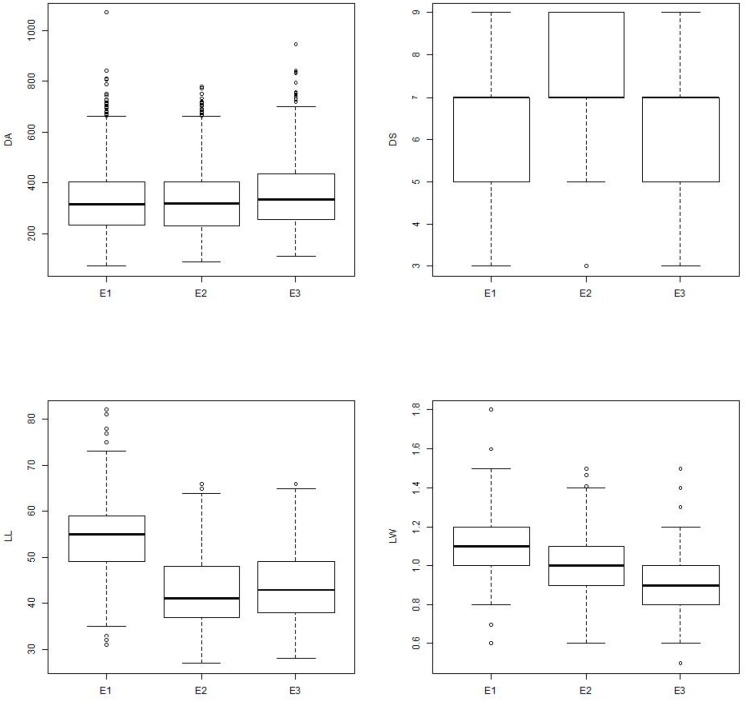
Boxplot showing the differences in parameters among different environments. Box edges represent the upper and lower quantile with median value shown as bold line in the middle of the box. Whiskers represent 1.5 times the quantile of the data. Individuals falling outside the range of the whiskers shown as open dot. E1 = Wet season 2013; E2 = Dry season 2013–2014; E3 = Wet season 2014; DA = Damaged area; DS = Damage score; LL = Leaf length; LW = Leaf width.

Among the environments, wet seasons were found most preferable to leaffolder phenotyping. Damage area showed similar pattern across three environments, however, variation in damage area and damage score was higher in wet season environments (E1 and E3) compared to dry season (E2). Among the two wet seasons, variation was higher in E1 compared to E3 which might be mainly due to the weather parameters. Temperature of 25–31°C and relative humidity of >80% are congenial for rice crop growth. During wet season 2013 (E1), temperature and relative humidity were optimum along with high rainfall compared to wet season 2014 (E3) that resulted in good crop growth and feeding by leaffolder causing more leaf area damage (Supplementary Table S6). Expression of leaf traits was better in E1 in comparison to other two environments (Figure 6). GGE biplot environment view revealed that all the three environments were representative of damage area and damage score traits with minor deviation from average environment axis. Wet season 2013 (E1) appeared closest to ideal test environment for damage score and found best for leaffolder phenotyping although dry season 2013–2014 (E2) emerged as ideal test environment for other three traits viz., damage area, LL and LW. Both wet season environments appeared more discriminative in case of damage area and LW followed by dry season while three environments were equally discriminative for damage score and LL. Yan and Tinker (2006) proposed that discriminating environments should be used as test environments as they gave more information on trait expression of genotypes. In case of damage area and LW all three environments came under same mega environment while for damage score both wet seasons were grouped into one mega environment and dry season came under a separate mega environment showing existence of seasonal specific adaptation. This information shows resistance and associated traits are influenced by environmental factors with G × E interactions and the minor variability observed is mainly caused by seasonal changes including weather parameters.

## Conclusion

In the present study, phenotyping of 160 RILs of a cross between a resistant parent, W1263 and a susceptible parent, TN1 using a rapid field screening method resulted in the identification of nine resistant RILs viz., MP 114, MP 215, MP 15, MP 217, MP 547, MP 108, MP 325, MP 120, and MP 27 which can be utilized as potential donors in leaffolder resistance breeding programs. AMMI and GGE biplot analysis revealed that two stable resistant lines G8(MP114) and G3(MP118) with lower damage score and damage area were promising for further yield evaluation trials. Alternatively, eight RILs were identified as highly susceptible which were at par with TN1 that can be used as susceptible checks in screening programs. Among the leaf morphological traits, leaffolder damage area showed a positive correlation with LW and negative correlation with LL. Precise demarcation of resistant and susceptible RILs in cluster analysis and appearance of all three environments under same mega environment for damage area and LW shows the robustness of our screening method. Continuous phenotypic frequency distributions revealed that the leaffolder resistance is quantitative in nature under polygenic control and damage area, damage score and LL showed very high broad-sense of heritability across three environments. Among the environments, both the wet seasons were found most preferable for phenotyping as compared to dry season. The G × E interactions clearly evidenced that the contribution of environment is minor and the resistance is majorly contributed by genotype main effect, however, seasonal variation influences the extent of trait expression. Further studies with crosses between identified stable resistant, moderately resistant and susceptible lines will be useful in genetic analysis of component traits and dissecting the mechanisms of leaffolder resistance.

## Ethics Statement

The authors declare that the experiments comply with the current laws of the country in which they were performed and in compliance with ethical standards.

## Author Contributions

PC conceived the work. PC, SN, GK planned the work. Field experiments for phenotypic evaluation were done by PC, SJ, TV, SM, and SL. DB and PC equally contributed during analysis of the data and writing the manuscript. All authors contributed for discussion and approved the manuscript.

## Conflict of Interest Statement

The authors declare that the research was conducted in the absence of any commercial or financial relationships that could be construed as a potential conflict of interest.

## Footnotes

1 http://imagej.nih.gov/ij/

2 http://darwin.cirad.fr/darwin/Home.php
