# Supplementary material for: Phenotyping and Genotype × Environment Interaction of Resistance to Leaffolder, Cnaphalocrocis medinalis Guenee (Lepidoptera: Pyralidae) in Rice
Source: Front Plant Sci. 2019 Feb 18;10:49. doi: 10.3389/fpls.2019.00049 (PMC6387916; doi:10.3389/fpls.2019.00049)
Supplement: Supplementary file 1 [file Table_1.DOC]

**Supplementary Table 1**. List of genotypes and environments and the codes used in AMMI and GGE analysis

| 1 | MP10 | G1 | 45 | MP209 | G45 | 89 | MP245 | G89 | 133 | MP451 | G133 |
| --- | --- | --- | --- | --- | --- | --- | --- | --- | --- | --- | --- |
| 2 | MP107 | G2 | 46 | MP21 | G46 | 90 | MP246 | G90 | 134 | MP453 | G134 |
| 3 | MP108 | G3 | 47 | MP211 | G47 | 91 | MP247 | G91 | 135 | MP457 | G135 |
| 4 | MP11 | G4 | 48 | MP212 | G48 | 92 | MP248 | G92 | 136 | MP459 | G136 |
| 5 | MP110 | G5 | 49 | MP215 | G49 | 93 | MP249 | G93 | 137 | MP460 | G137 |
| 6 | MP111 | G6 | 50 | MP216 | G50 | 94 | MP300 | G94 | 138 | MP520 | G138 |
| 7 | MP112 | G7 | 51 | MP217 | G51 | 95 | MP301 | G95 | 139 | MP524 | G139 |
| 8 | MP114 | G8 | 52 | MP22 | G52 | 96 | MP303 | G96 | 140 | MP527 | G140 |
| 9 | MP115 | G9 | 53 | MP23 | G53 | 97 | MP307 | G97 | 141 | MP528 | G141 |
| 10 | MP116 | G10 | 54 | MP27 | G54 | 98 | MP312 | G98 | 142 | MP530 | G142 |
| 11 | MP117 | G11 | 55 | MP28 | G55 | 99 | MP313 | G99 | 143 | MP531 | G143 |
| 12 | MP12 | G12 | 56 | MP31 | G56 | 100 | MP314 | G100 | 144 | MP533 | G144 |
| 13 | MP120 | G13 | 57 | MP32 | G57 | 101 | MP316 | G101 | 145 | MP536 | G145 |
| 14 | MP121 | G14 | 58 | MP35 | G58 | 102 | MP317 | G102 | 146 | MP537 | G146 |
| 15 | MP122 | G15 | 59 | MP37 | G59 | 103 | MP318 | G103 | 147 | MP539 | G147 |
| 16 | MP123 | G16 | 60 | MP4 | G60 | 104 | MP320 | G104 | 148 | MP540 | G148 |
| 17 | MP124 | G17 | 61 | MP40 | G61 | 105 | MP323 | G105 | 149 | MP541 | G149 |
| 18 | MP125 | G18 | 62 | MP42 | G62 | 106 | MP324 | G106 | 150 | MP542 | G150 |
| 19 | MP126 | G19 | 63 | MP44 | G63 | 107 | MP325 | G107 | 151 | MP543 | G151 |
| 20 | MP127 | G20 | 64 | MP45 | G64 | 108 | MP327 | G108 | 152 | MP544 | G152 |
| 21 | MP131 | G21 | 65 | MP46 | G65 | 109 | MP331 | G109 | 153 | MP546 | G153 |
| 22 | MP132 | G22 | 66 | MP7 | G66 | 110 | MP337 | G110 | 154 | MP547 | G154 |
| 23 | MP133 | G23 | 67 | MP8 | G67 | 111 | MP338 | G111 | 155 | MP549 | G155 |
| 24 | MP134 | G24 | 68 | MP9 | G68 | 112 | MP339 | G112 | 156 | MP551 | G156 |
| 25 | MP135 | G25 | 69 | MP220 | G69 | 113 | MP340 | G113 | 157 | MP553 | G157 |
| 26 | MP136 | G26 | 70 | MP221 | G70 | 114 | MP342 | G114 | 158 | MP555 | G158 |
| 27 | MP138 | G27 | 71 | MP222 | G71 | 115 | MP344 | G115 | 159 | MP556 | G159 |
| 28 | MP139 | G28 | 72 | MP223 | G72 | 116 | MP348 | G116 | 160 | MP558 | G160 |
| 29 | MP14 | G29 | 73 | MP224 | G73 | 117 | MP352 | G117 | 161 | TN | 1 G161 |
| 30 | MP142 | G30 | 74 | MP226 | G74 | 118 | MP353 | G118 | 162 | W126 | 3 G162 |
| 31 | MP143 | G31 | 75 | MP227 | G75 | 119 | MP355 | G119 |  |  |  |
| 32 | MP144 | G32 | 76 | MP228 | G76 | 120 | MP357 | G120 | Environment | | Code |
| 33 | MP145 | G33 | 77 | MP230 | G77 | 121 | MP361 | G121 | 1 | E1 | E1 |
| 34 | MP146 | G34 | 78 | MP231 | G78 | 122 | MP425 | G122 | 2 | E2 | E2 |
| 35 | MP148 | G35 | 79 | MP232 | G79 | 123 | MP434 | G123 | 3 | E3 | E3 |
| 36 | MP149 | G36 | 80 | MP233 | G80 | 124 | MP435 | G124 |  |  |  |
| 37 | MP15 | G37 | 81 | MP234 | G81 | 125 | MP436 | G125 |  |  |  |
| 38 | MP16 | G38 | 82 | MP235 | G82 | 126 | MP439 | G126 |  |  |  |
| 39 | MP17 | G39 | 83 | MP236 | G83 | 127 | MP440 | G127 |  |  |  |
| 40 | MP18 | G40 | 84 | MP237 | G84 | 128 | MP442 | G128 |  |  |  |
| 41 | MP19 | G41 | 85 | MP240 | G85 | 129 | MP443 | G129 |  |  |  |
| 42 | MP2 | G42 | 86 | MP241 | G86 | 130 | MP444 | G130 |  |  |  |
| 43 | MP20 | G43 | 87 | MP243 | G87 | 131 | MP445 | G131 |  |  |  |
| 44 | MP206 | G44 | 88 | MP244 | G88 | 132 | MP448 | G132 |  |  |  |
