# Supplementary material for: Phenotyping and Genotype × Environment Interaction of Resistance to Leaffolder, Cnaphalocrocis medinalis Guenee (Lepidoptera: Pyralidae) in Rice
Source: Front Plant Sci. 2019 Feb 18;10:49. doi: 10.3389/fpls.2019.00049 (PMC6387916; doi:10.3389/fpls.2019.00049)
Supplement: Supplementary file 2 [file Table_2.DOC]

**Supplementary Table 2. Principal components for genotypes on damage area and damage score in 160 RILs and parents**

| Levels | type | DA | PC1 | PC2 |  | Levels | type | DS | PC1 | PC2 |
| --- | --- | --- | --- | --- | --- | --- | --- | --- | --- | --- |
| G8 | GEN | 112.18 | -402.18 | 11.79 |  | G8 | GEN | 3.00 | -6.27 | 0.71 |
| G37 | GEN | 124.98 | -379.89 | -13.97 |  | G162 | GEN | 3.22 | -5.89 | 0.26 |
| G49 | GEN | 142.86 | -348.40 | -10.38 |  | G49 | GEN | 3.22 | -5.89 | 0.26 |
| G3 | GEN | 146.83 | -340.80 | 9.44 |  | G37 | GEN | 3.44 | -5.52 | -0.18 |
| G107 | GEN | 150.76 | -334.71 | -42.15 |  | G51 | GEN | 3.44 | -5.52 | -0.18 |
| G12 | GEN | 158.55 | -319.68 | -43.93 |  | G154 | GEN | 3.44 | -5.50 | 0.75 |
| G54 | GEN | 163.85 | -312.31 | -40.79 |  | G3 | GEN | 3.44 | -5.49 | 1.14 |
| G13 | GEN | 166.38 | -307.86 | -33.29 |  | G107 | GEN | 3.67 | -5.13 | -0.24 |
| G51 | GEN | 167.43 | -305.89 | -3.25 |  | G13 | GEN | 3.67 | -5.13 | -0.24 |
| G162 | GEN | 169.89 | -300.99 | -18.32 |  | G54 | GEN | 3.67 | -5.13 | -0.24 |
| G20 | GEN | 173.58 | -295.83 | 1.41 |  | G12 | GEN | 4.11 | -4.36 | -0.75 |
| G148 | GEN | 176.16 | -290.86 | -30.44 |  | G148 | GEN | 4.11 | -4.36 | -0.20 |
| G154 | GEN | 176.78 | -290.52 | -1.77 |  | G20 | GEN | 4.11 | -4.36 | -0.20 |
| G43 | GEN | 183.22 | -278.93 | -15.97 |  | G71 | GEN | 4.11 | -4.36 | 0.35 |
| G71 | GEN | 184.06 | -277.95 | 18.65 |  | G43 | GEN | 4.11 | -4.35 | 0.19 |
| G108 | GEN | 191.27 | -265.23 | -37.55 |  | G1 | GEN | 4.33 | -3.97 | 0.84 |
| G136 | GEN | 193.80 | -259.91 | 5.01 |  | G152 | GEN | 4.33 | -3.97 | 0.84 |
| G21 | GEN | 195.81 | -256.89 | -15.26 |  | G21 | GEN | 4.56 | -3.58 | -0.32 |
| G135 | GEN | 200.91 | -248.14 | 14.82 |  | G146 | GEN | 4.56 | -3.58 | 0.23 |
| G34 | GEN | 202.31 | -245.63 | 30.56 |  | G34 | GEN | 4.56 | -3.58 | 0.78 |
| G1 | GEN | 204.95 | -242.20 | 64.34 |  | G108 | GEN | 4.78 | -3.21 | -0.22 |
| G96 | GEN | 205.44 | -240.93 | -14.17 |  | G135 | GEN | 4.78 | -3.19 | 0.72 |
| G152 | GEN | 206.60 | -239.54 | 18.30 |  | G136 | GEN | 4.78 | -3.19 | 0.72 |
| G146 | GEN | 207.72 | -236.87 | -3.87 |  | G42 | GEN | 4.78 | -3.19 | 0.72 |
| G15 | GEN | 208.46 | -235.26 | 5.92 |  | G52 | GEN | 4.78 | -3.19 | 0.72 |
| G42 | GEN | 208.92 | -234.27 | 9.88 |  | G15 | GEN | 5.00 | -2.82 | -0.28 |
| G52 | GEN | 214.29 | -224.84 | 30.03 |  | G75 | GEN | 5.00 | -2.82 | 0.27 |
| G137 | GEN | 223.31 | -209.01 | -10.77 |  | G96 | GEN | 5.00 | -2.82 | 0.27 |
| G78 | GEN | 231.12 | -195.96 | 11.33 |  | G103 | GEN | 5.00 | -2.80 | 0.66 |
| G75 | GEN | 231.97 | -194.78 | 9.70 |  | G137 | GEN | 5.00 | -2.80 | 0.66 |
| G79 | GEN | 240.21 | -180.17 | 33.23 |  | G79 | GEN | 5.22 | -2.44 | -0.17 |
| G103 | GEN | 246.23 | -168.85 | -10.60 |  | G16 | GEN | 5.22 | -2.44 | 0.38 |
| G88 | GEN | 247.17 | -168.62 | 10.67 |  | G78 | GEN | 5.22 | -2.43 | 0.21 |
| G101 | GEN | 247.22 | -168.30 | -34.23 |  | G106 | GEN | 5.44 | -2.05 | -0.23 |
| G30 | GEN | 248.02 | -166.89 | 0.42 |  | G131 | GEN | 5.44 | -2.05 | -0.23 |
| G125 | GEN | 249.02 | -166.48 | -18.13 |  | G24 | GEN | 5.44 | -2.04 | 0.15 |
| G73 | GEN | 248.73 | -166.41 | -68.61 |  | G101 | GEN | 5.67 | -1.68 | -0.68 |
| G131 | GEN | 252.38 | -158.50 | -11.44 |  | G30 | GEN | 5.67 | -1.68 | -0.68 |
| G57 | GEN | 252.60 | -158.26 | -9.64 |  | G57 | GEN | 5.67 | -1.68 | -0.68 |
| G24 | GEN | 253.24 | -156.54 | -28.78 |  | G7 | GEN | 5.67 | -1.68 | -0.68 |
| G106 | GEN | 253.68 | -156.29 | 6.12 |  | G90 | GEN | 5.67 | -1.68 | -0.68 |
| G16 | GEN | 256.79 | -152.56 | 52.03 |  | G97 | GEN | 5.67 | -1.68 | -0.68 |
| G69 | GEN | 263.09 | -140.09 | -7.74 |  | G19 | GEN | 5.67 | -1.66 | 0.26 |
| G90 | GEN | 263.71 | -139.33 | 18.16 |  | G88 | GEN | 5.67 | -1.66 | 0.26 |
| G104 | GEN | 264.00 | -138.85 | -35.55 |  | G9 | GEN | 5.67 | -1.66 | 0.26 |
| G97 | GEN | 265.24 | -137.40 | -0.15 |  | G39 | GEN | 5.67 | -1.65 | 1.19 |
| G7 | GEN | 265.11 | -137.00 | -34.03 |  | G40 | GEN | 5.67 | -1.63 | 2.13 |
| G159 | GEN | 266.06 | -136.90 | -20.78 |  | G159 | GEN | 5.89 | -1.30 | -1.13 |
| G38 | GEN | 265.84 | -135.42 | 51.60 |  | G73 | GEN | 5.89 | -1.30 | -1.13 |
| G77 | GEN | 265.93 | -135.11 | -25.00 |  | G104 | GEN | 5.89 | -1.29 | -0.74 |
| G40 | GEN | 268.14 | -130.64 | 65.84 |  | G130 | GEN | 5.89 | -1.29 | -0.74 |
| G29 | GEN | 269.41 | -129.57 | -7.98 |  | G160 | GEN | 5.89 | -1.29 | -0.74 |
| G50 | GEN | 270.22 | -129.48 | -0.30 |  | G29 | GEN | 5.89 | -1.29 | -0.74 |
| G122 | GEN | 274.52 | -121.01 | 4.08 |  | G47 | GEN | 5.89 | -1.29 | -0.74 |
| G105 | GEN | 275.04 | -120.37 | -1.32 |  | G69 | GEN | 5.89 | -1.29 | -0.74 |
| G9 | GEN | 276.00 | -118.02 | 7.83 |  | G105 | GEN | 5.89 | -1.29 | -0.19 |
| G72 | GEN | 276.30 | -117.95 | -61.82 |  | G122 | GEN | 5.89 | -1.29 | -0.19 |
| G94 | GEN | 278.60 | -113.53 | 18.94 |  | G50 | GEN | 5.89 | -1.29 | -0.19 |
| G82 | GEN | 279.52 | -112.19 | -45.41 |  | G77 | GEN | 5.89 | -1.29 | -0.19 |
| G160 | GEN | 279.16 | -112.04 | -8.10 |  | G32 | GEN | 5.89 | -1.27 | 0.19 |
| G32 | GEN | 281.27 | -108.60 | -1.72 |  | G38 | GEN | 5.89 | -1.27 | 0.75 |
| G19 | GEN | 283.83 | -104.66 | 7.91 |  | G94 | GEN | 5.89 | -1.27 | 0.75 |
| G47 | GEN | 283.83 | -104.60 | -34.21 |  | G72 | GEN | 6.11 | -0.91 | -1.19 |
| G130 | GEN | 284.92 | -102.28 | -30.54 |  | G82 | GEN | 6.11 | -0.91 | -1.19 |
| G39 | GEN | 298.40 | -79.66 | 32.11 |  | G27 | GEN | 6.11 | -0.91 | -0.64 |
| G99 | GEN | 297.51 | -79.09 | -81.96 |  | G67 | GEN | 6.33 | -0.52 | -0.15 |
| G23 | GEN | 301.59 | -73.33 | 30.55 |  | G99 | GEN | 6.33 | -0.51 | -0.87 |
| G27 | GEN | 305.44 | -67.76 | -31.22 |  | G125 | GEN | 6.33 | -0.51 | 0.79 |
| G119 | GEN | 308.05 | -63.12 | 22.62 |  | G23 | GEN | 6.33 | -0.49 | 1.18 |
| G124 | GEN | 314.09 | -52.99 | 46.74 |  | G4 | GEN | 6.33 | -0.46 | 3.05 |
| G4 | GEN | 314.67 | -48.63 | 218.60 |  | G74 | GEN | 6.56 | -0.13 | -0.76 |
| G121 | GEN | 319.38 | -44.07 | -2.88 |  | G6 | GEN | 6.56 | -0.13 | -0.21 |
| G66 | GEN | 320.57 | -43.60 | 259.25 |  | G66 | GEN | 6.56 | -0.13 | 2.00 |
| G67 | GEN | 321.53 | -40.63 | 85.06 |  | G31 | GEN | 6.56 | -0.12 | 0.18 |
| G44 | GEN | 321.28 | -40.12 | -26.50 |  | G124 | GEN | 6.56 | -0.12 | 0.73 |
| G87 | GEN | 321.57 | -39.72 | -1.24 |  | G70 | GEN | 6.56 | -0.12 | 0.73 |
| G74 | GEN | 321.39 | -39.37 | -19.80 |  | G44 | GEN | 6.56 | -0.11 | 0.01 |
| G113 | GEN | 321.31 | -38.07 | 6.59 |  | G60 | GEN | 6.78 | 0.24 | -2.31 |
| G158 | GEN | 324.26 | -34.06 | 31.61 |  | G83 | GEN | 6.78 | 0.24 | -1.21 |
| G36 | GEN | 324.85 | -33.36 | 17.58 |  | G93 | GEN | 6.78 | 0.24 | -0.66 |
| G70 | GEN | 326.43 | -31.25 | 25.34 |  | G119 | GEN | 6.78 | 0.26 | 0.28 |
| G31 | GEN | 327.43 | -28.82 | -17.04 |  | G134 | GEN | 6.78 | 0.26 | 0.28 |
| G93 | GEN | 329.77 | -25.90 | -12.86 |  | G36 | GEN | 6.78 | 0.26 | 0.28 |
| G134 | GEN | 331.10 | -23.00 | 26.32 |  | G17 | GEN | 7.00 | 0.62 | -1.65 |
| G83 | GEN | 330.83 | -22.82 | -25.56 |  | G5 | GEN | 7.00 | 0.62 | -1.10 |
| G45 | GEN | 332.22 | -20.72 | -15.65 |  | G45 | GEN | 7.00 | 0.63 | -0.72 |
| G116 | GEN | 334.52 | -17.31 | -74.04 |  | G126 | GEN | 7.00 | 0.64 | -0.33 |
| G60 | GEN | 335.32 | -15.42 | -140.45 |  | G138 | GEN | 7.00 | 0.65 | 0.22 |
| G5 | GEN | 336.39 | -14.94 | -22.16 |  | G46 | GEN | 7.00 | 0.65 | 0.22 |
| G80 | GEN | 336.51 | -13.98 | -21.76 |  | G87 | GEN | 7.00 | 0.65 | 0.22 |
| G10 | GEN | 337.67 | -12.19 | -0.91 |  | G117 | GEN | 7.00 | 0.65 | 0.77 |
| G46 | GEN | 338.45 | -9.92 | 1.98 |  | G113 | GEN | 7.00 | 0.66 | 0.61 |
| G6 | GEN | 343.36 | -2.65 | -2.08 |  | G68 | GEN | 7.00 | 0.68 | 2.10 |
| G138 | GEN | 344.04 | -0.21 | 46.53 |  | G116 | GEN | 7.22 | 1.00 | -1.72 |
| G76 | GEN | 345.58 | 1.37 | -8.42 |  | G121 | GEN | 7.22 | 1.01 | -0.61 |
| G126 | GEN | 347.17 | 5.78 | -51.32 |  | G80 | GEN | 7.22 | 1.01 | -0.61 |
| G115 | GEN | 352.25 | 14.89 | -23.33 |  | G10 | GEN | 7.22 | 1.02 | -0.23 |
| G117 | GEN | 353.38 | 16.21 | 79.81 |  | G22 | GEN | 7.22 | 1.02 | -0.23 |
| G143 | GEN | 355.01 | 19.03 | -43.26 |  | G115 | GEN | 7.22 | 1.03 | 0.16 |
| G17 | GEN | 357.99 | 22.87 | -56.87 |  | G151 | GEN | 7.22 | 1.03 | 0.16 |
| G22 | GEN | 359.48 | 25.08 | -23.42 |  | G62 | GEN | 7.22 | 1.03 | 0.16 |
| G127 | GEN | 360.15 | 27.37 | -13.05 |  | G139 | GEN | 7.44 | 1.40 | -0.67 |
| G68 | GEN | 359.27 | 28.00 | 120.73 |  | G76 | GEN | 7.44 | 1.40 | -0.67 |
| G111 | GEN | 360.21 | 28.10 | -24.81 |  | G127 | GEN | 7.44 | 1.41 | -0.29 |
| G151 | GEN | 362.50 | 31.27 | -7.17 |  | G142 | GEN | 7.44 | 1.41 | -0.29 |
| G114 | GEN | 362.50 | 31.63 | -12.40 |  | G143 | GEN | 7.44 | 1.41 | -0.29 |
| G91 | GEN | 363.89 | 33.49 | -44.65 |  | G150 | GEN | 7.44 | 1.41 | -0.29 |
| G112 | GEN | 364.88 | 35.20 | -47.90 |  | G65 | GEN | 7.44 | 1.44 | 1.59 |
| G139 | GEN | 364.90 | 35.44 | 8.26 |  | G102 | GEN | 7.67 | 1.79 | -0.73 |
| G110 | GEN | 365.22 | 35.65 | -32.12 |  | G109 | GEN | 7.67 | 1.79 | -0.73 |
| G62 | GEN | 368.95 | 43.46 | 59.24 |  | G110 | GEN | 7.67 | 1.79 | -0.73 |
| G142 | GEN | 372.49 | 48.79 | 10.07 |  | G111 | GEN | 7.67 | 1.79 | -0.73 |
| G150 | GEN | 375.05 | 53.71 | -37.75 |  | G112 | GEN | 7.67 | 1.79 | -0.73 |
| G147 | GEN | 379.81 | 62.19 | -20.62 |  | G114 | GEN | 7.67 | 1.79 | -0.73 |
| G109 | GEN | 382.53 | 65.95 | 10.95 |  | G129 | GEN | 7.67 | 1.79 | -0.73 |
| G129 | GEN | 385.42 | 71.33 | -33.18 |  | G133 | GEN | 7.67 | 1.79 | -0.73 |
| G28 | GEN | 393.74 | 85.79 | 59.15 |  | G147 | GEN | 7.67 | 1.79 | -0.73 |
| G63 | GEN | 396.52 | 90.92 | -50.18 |  | G155 | GEN | 7.67 | 1.79 | -0.73 |
| G118 | GEN | 397.33 | 91.86 | 65.34 |  | G48 | GEN | 7.67 | 1.79 | -0.73 |
| G102 | GEN | 405.97 | 107.78 | -11.73 |  | G63 | GEN | 7.67 | 1.79 | -0.73 |
| G133 | GEN | 407.57 | 107.95 | -25.42 |  | G91 | GEN | 7.67 | 1.79 | -0.73 |
| G14 | GEN | 408.31 | 111.12 | 29.34 |  | G28 | GEN | 7.67 | 1.80 | 0.20 |
| G18 | GEN | 415.60 | 123.80 | 8.06 |  | G158 | GEN | 7.67 | 1.81 | 0.04 |
| G155 | GEN | 417.48 | 126.44 | -0.25 |  | G18 | GEN | 7.89 | 2.17 | -0.80 |
| G11 | GEN | 418.89 | 131.07 | -36.50 |  | G81 | GEN | 7.89 | 2.17 | -0.80 |
| G84 | GEN | 422.14 | 134.67 | -15.69 |  | G118 | GEN | 7.89 | 2.18 | -0.24 |
| G56 | GEN | 426.43 | 142.06 | 13.14 |  | G14 | GEN | 7.89 | 2.18 | -0.24 |
| G95 | GEN | 427.02 | 142.37 | 6.02 |  | G145 | GEN | 7.89 | 2.18 | -0.24 |
| G65 | GEN | 426.67 | 142.97 | 40.12 |  | G95 | GEN | 7.89 | 2.18 | -0.24 |
| G26 | GEN | 432.16 | 154.11 | 74.37 |  | G26 | GEN | 7.89 | 2.20 | 1.08 |
| G64 | GEN | 435.16 | 157.80 | 41.55 |  | G11 | GEN | 8.11 | 2.56 | -0.86 |
| G156 | GEN | 435.38 | 158.52 | 50.30 |  | G156 | GEN | 8.11 | 2.56 | -0.30 |
| G92 | GEN | 439.07 | 164.41 | 16.22 |  | G35 | GEN | 8.11 | 2.56 | -0.30 |
| G145 | GEN | 442.71 | 168.91 | -3.38 |  | G56 | GEN | 8.11 | 2.56 | -0.30 |
| G48 | GEN | 443.77 | 172.28 | -8.18 |  | G84 | GEN | 8.11 | 2.56 | -0.30 |
| G61 | GEN | 447.50 | 179.01 | -48.19 |  | G92 | GEN | 8.11 | 2.56 | -0.30 |
| G81 | GEN | 453.29 | 188.21 | -35.42 |  | G41 | GEN | 8.11 | 2.57 | 0.25 |
| G41 | GEN | 456.23 | 194.21 | 46.39 |  | G61 | GEN | 8.33 | 2.95 | -0.37 |
| G53 | GEN | 457.76 | 195.79 | 24.49 |  | G149 | GEN | 8.33 | 2.96 | 0.74 |
| G89 | GEN | 471.58 | 221.06 | 99.54 |  | G53 | GEN | 8.33 | 2.96 | 0.74 |
| G35 | GEN | 478.45 | 230.97 | -46.84 |  | G58 | GEN | 8.56 | 3.34 | 0.68 |
| G58 | GEN | 497.93 | 263.44 | -24.63 |  | G64 | GEN | 8.56 | 3.34 | 0.68 |
| G149 | GEN | 497.64 | 265.27 | 107.38 |  | G89 | GEN | 8.56 | 3.34 | 0.68 |
| G132 | GEN | 508.01 | 284.32 | -1.86 |  | G100 | GEN | 9.00 | 4.12 | 0.55 |
| G120 | GEN | 514.34 | 294.69 | -26.90 |  | G120 | GEN | 9.00 | 4.12 | 0.55 |
| G55 | GEN | 524.14 | 312.58 | -31.64 |  | G123 | GEN | 9.00 | 4.12 | 0.55 |
| G85 | GEN | 528.08 | 318.41 | -47.00 |  | G128 | GEN | 9.00 | 4.12 | 0.55 |
| G153 | GEN | 536.09 | 334.08 | 77.23 |  | G132 | GEN | 9.00 | 4.12 | 0.55 |
| G161 | GEN | 550.46 | 360.29 | 37.16 |  | G140 | GEN | 9.00 | 4.12 | 0.55 |
| G25 | GEN | 562.74 | 379.00 | -77.60 |  | G141 | GEN | 9.00 | 4.12 | 0.55 |
| G86 | GEN | 563.25 | 381.83 | 130.16 |  | G144 | GEN | 9.00 | 4.12 | 0.55 |
| G2 | GEN | 584.74 | 418.07 | -130.35 |  | G153 | GEN | 9.00 | 4.12 | 0.55 |
| G140 | GEN | 613.21 | 466.66 | -83.05 |  | G157 | GEN | 9.00 | 4.12 | 0.55 |
| G100 | GEN | 639.88 | 511.50 | -6.62 |  | G161 | GEN | 9.00 | 4.12 | 0.55 |
| G98 | GEN | 644.42 | 520.10 | -32.64 |  | G2 | GEN | 9.00 | 4.12 | 0.55 |
| G157 | GEN | 666.96 | 559.08 | 35.96 |  | G25 | GEN | 9.00 | 4.12 | 0.55 |
| G144 | GEN | 689.91 | 599.41 | -66.89 |  | G33 | GEN | 9.00 | 4.12 | 0.55 |
| G123 | GEN | 701.76 | 618.61 | -12.48 |  | G55 | GEN | 9.00 | 4.12 | 0.55 |
| G128 | GEN | 712.79 | 637.31 | 44.22 |  | G59 | GEN | 9.00 | 4.12 | 0.55 |
| G33 | GEN | 722.55 | 656.69 | -56.55 |  | G85 | GEN | 9.00 | 4.12 | 0.55 |
| G59 | GEN | 729.67 | 667.62 | 31.86 |  | G86 | GEN | 9.00 | 4.12 | 0.55 |
| G141 | GEN | 780.51 | 756.06 | -7.59 |  | G98 | GEN | 9.00 | 4.12 | 0.55 |
| E1 | ENV | 338.32 | 0.59 | -0.48 |  | E1 | ENV | 6.12 | 0.58 | -0.09 |
| E2 | ENV | 335.99 | 0.56 | -0.33 |  | E2 | ENV | 7.37 | 0.56 | -0.67 |
| E3 | ENV | 358.34 | 0.58 | 0.81 |  | E3 | ENV | 6.40 | 0.59 | 0.74 |
