# Supplementary material for: Phenotyping and Genotype × Environment Interaction of Resistance to Leaffolder, Cnaphalocrocis medinalis Guenee (Lepidoptera: Pyralidae) in Rice
Source: Front Plant Sci. 2019 Feb 18;10:49. doi: 10.3389/fpls.2019.00049 (PMC6387916; doi:10.3389/fpls.2019.00049)
Supplement: Supplementary file 3 [file Table_3.DOC]

**Supplementary Table 3. Principal components for genotypes on leaf length and leaf width in 160 RILs and parents**

| Levels | type | LL | PC1 | PC2 |  | Levels | type | LW | PC1 | PC2 |
| --- | --- | --- | --- | --- | --- | --- | --- | --- | --- | --- |
| G15 | GEN | 35.11 | -20.64 | 8.47 |  | G161 | GEN | 1.43 | -0.67 | -0.10 |
| G161 | GEN | 35.26 | -20.34 | 6.56 |  | G61 | GEN | 1.24 | -0.43 | -0.08 |
| G122 | GEN | 35.44 | -19.96 | 7.07 |  | G76 | GEN | 1.26 | -0.40 | -0.08 |
| G46 | GEN | 36.11 | -18.76 | 0.79 |  | G63 | GEN | 1.23 | -0.38 | -0.11 |
| G57 | GEN | 37.00 | -17.01 | 3.06 |  | G141 | GEN | 1.26 | -0.36 | -0.05 |
| G11 | GEN | 37.33 | -16.88 | 7.04 |  | G144 | GEN | 1.24 | -0.34 | -0.05 |
| G41 | GEN | 37.33 | -16.62 | -7.59 |  | G68 | GEN | 1.22 | -0.34 | 0.11 |
| G69 | GEN | 37.33 | -16.46 | -4.10 |  | G75 | GEN | 1.20 | -0.31 | -0.08 |
| G23 | GEN | 38.33 | -15.08 | 5.33 |  | G79 | GEN | 1.18 | -0.29 | 0.09 |
| G25 | GEN | 38.44 | -14.52 | -6.09 |  | G151 | GEN | 1.22 | -0.28 | -0.12 |
| G16 | GEN | 38.67 | -14.50 | 6.89 |  | G48 | GEN | 1.13 | -0.23 | -0.09 |
| G115 | GEN | 38.67 | -14.23 | -0.96 |  | G1 | GEN | 1.16 | -0.23 | -0.15 |
| G90 | GEN | 38.89 | -14.06 | 6.72 |  | G14 | GEN | 1.13 | -0.22 | -0.12 |
| G151 | GEN | 38.89 | -13.76 | -7.57 |  | G67 | GEN | 1.14 | -0.22 | -0.15 |
| G121 | GEN | 39.22 | -13.32 | 0.09 |  | G77 | GEN | 1.16 | -0.22 | -0.05 |
| G76 | GEN | 39.56 | -12.95 | 2.05 |  | G12 | GEN | 1.14 | -0.21 | -0.06 |
| G94 | GEN | 39.44 | -12.72 | -1.43 |  | G72 | GEN | 1.13 | -0.20 | 0.17 |
| G111 | GEN | 39.56 | -12.65 | -6.54 |  | G11 | GEN | 1.12 | -0.20 | -0.10 |
| G123 | GEN | 39.78 | -12.42 | -3.42 |  | G29 | GEN | 1.12 | -0.20 | -0.10 |
| G104 | GEN | 40.22 | -11.65 | 10.66 |  | G23 | GEN | 1.16 | -0.20 | -0.02 |
| G141 | GEN | 40.22 | -11.52 | -6.45 |  | G40 | GEN | 1.12 | -0.19 | 0.17 |
| G98 | GEN | 40.44 | -11.13 | -2.05 |  | G4 | GEN | 1.14 | -0.19 | -0.10 |
| G5 | GEN | 40.89 | -10.79 | 14.65 |  | G121 | GEN | 1.10 | -0.17 | 0.22 |
| G95 | GEN | 40.67 | -10.65 | 0.53 |  | G27 | GEN | 1.11 | -0.17 | -0.07 |
| G96 | GEN | 41.22 | -9.90 | 6.48 |  | G116 | GEN | 1.13 | -0.16 | -0.04 |
| G149 | GEN | 41.44 | -9.27 | -8.32 |  | G64 | GEN | 1.13 | -0.16 | -0.04 |
| G56 | GEN | 41.56 | -9.17 | -8.27 |  | G98 | GEN | 1.11 | -0.15 | 0.04 |
| G144 | GEN | 41.56 | -9.15 | -8.34 |  | G62 | GEN | 1.11 | -0.15 | 0.01 |
| G118 | GEN | 41.89 | -9.13 | 11.60 |  | G78 | GEN | 1.11 | -0.15 | 0.01 |
| G24 | GEN | 42.00 | -8.68 | 3.13 |  | G54 | GEN | 1.10 | -0.14 | -0.13 |
| G14 | GEN | 42.11 | -8.67 | 10.49 |  | G112 | GEN | 1.12 | -0.14 | 0.29 |
| G88 | GEN | 42.22 | -8.51 | 6.65 |  | G89 | GEN | 1.10 | -0.14 | -0.08 |
| G43 | GEN | 42.00 | -8.37 | -8.34 |  | G92 | GEN | 1.10 | -0.13 | 0.01 |
| G148 | GEN | 42.11 | -8.27 | -8.29 |  | G58 | GEN | 1.07 | -0.13 | 0.16 |
| G129 | GEN | 42.00 | -8.20 | -8.21 |  | G157 | GEN | 1.10 | -0.12 | 0.03 |
| G30 | GEN | 42.44 | -8.09 | 9.64 |  | G85 | GEN | 1.08 | -0.12 | -0.04 |
| G160 | GEN | 42.22 | -8.08 | -8.04 |  | G9 | GEN | 1.09 | -0.12 | 0.11 |
| G82 | GEN | 42.78 | -7.47 | 2.47 |  | G118 | GEN | 1.09 | -0.11 | 0.20 |
| G131 | GEN | 42.67 | -7.31 | -7.17 |  | G52 | GEN | 1.09 | -0.11 | 0.20 |
| G44 | GEN | 42.89 | -6.98 | 2.92 |  | G106 | GEN | 1.09 | -0.11 | -0.09 |
| G53 | GEN | 43.11 | -6.51 | -8.12 |  | G97 | GEN | 1.10 | -0.10 | 0.10 |
| G77 | GEN | 43.22 | -6.45 | 0.65 |  | G47 | GEN | 1.07 | -0.10 | 0.11 |
| G65 | GEN | 43.44 | -6.21 | 2.83 |  | G83 | GEN | 1.08 | -0.09 | -0.04 |
| G32 | GEN | 43.44 | -6.05 | 2.16 |  | G88 | GEN | 1.08 | -0.09 | 0.01 |
| G102 | GEN | 43.56 | -5.94 | -0.67 |  | G154 | GEN | 1.08 | -0.08 | 0.14 |
| G54 | GEN | 43.67 | -5.91 | 7.29 |  | G33 | GEN | 1.07 | -0.08 | 0.01 |
| G8 | GEN | 43.67 | -5.70 | 4.67 |  | G50 | GEN | 1.06 | -0.07 | 0.05 |
| G45 | GEN | 43.67 | -5.50 | -6.46 |  | G104 | GEN | 1.07 | -0.06 | -0.14 |
| G139 | GEN | 43.78 | -5.34 | -5.94 |  | G45 | GEN | 1.06 | -0.06 | 0.02 |
| G130 | GEN | 44.00 | -5.27 | -6.31 |  | G101 | GEN | 1.07 | -0.06 | -0.01 |
| G162 | GEN | 44.04 | -5.13 | 4.01 |  | G16 | GEN | 1.08 | -0.06 | -0.12 |
| G138 | GEN | 44.11 | -4.91 | -4.39 |  | G109 | GEN | 1.06 | -0.06 | -0.01 |
| G112 | GEN | 44.22 | -4.79 | 2.44 |  | G57 | GEN | 1.07 | -0.05 | 0.13 |
| G135 | GEN | 44.22 | -4.78 | 1.97 |  | G80 | GEN | 1.06 | -0.05 | 0.12 |
| G72 | GEN | 44.78 | -3.88 | 10.73 |  | G51 | GEN | 1.02 | -0.05 | 0.22 |
| G34 | GEN | 44.78 | -3.80 | -0.27 |  | G86 | GEN | 1.04 | -0.04 | -0.09 |
| G12 | GEN | 44.78 | -3.76 | -4.56 |  | G103 | GEN | 1.04 | -0.03 | -0.04 |
| G157 | GEN | 44.78 | -3.55 | -9.80 |  | G105 | GEN | 1.04 | -0.03 | -0.04 |
| G136 | GEN | 44.89 | -3.49 | -3.91 |  | G15 | GEN | 1.06 | -0.03 | -0.15 |
| G137 | GEN | 45.11 | -3.45 | 4.18 |  | G159 | GEN | 1.06 | -0.03 | 0.11 |
| G117 | GEN | 45.00 | -3.45 | 4.46 |  | G140 | GEN | 1.06 | -0.03 | -0.11 |
| G127 | GEN | 45.00 | -3.40 | -3.59 |  | G134 | GEN | 1.04 | -0.03 | 0.05 |
| G66 | GEN | 45.11 | -3.04 | -3.88 |  | G41 | GEN | 1.03 | -0.03 | 0.21 |
| G18 | GEN | 45.44 | -2.99 | 15.53 |  | G59 | GEN | 1.04 | -0.03 | -0.12 |
| G49 | GEN | 45.56 | -2.64 | 14.84 |  | G65 | GEN | 1.04 | -0.03 | -0.12 |
| G83 | GEN | 45.33 | -2.62 | -5.73 |  | G153 | GEN | 1.06 | -0.03 | 0.03 |
| G97 | GEN | 45.67 | -2.59 | 8.45 |  | G117 | GEN | 1.07 | -0.02 | -0.04 |
| G81 | GEN | 45.56 | -2.35 | -2.74 |  | G18 | GEN | 1.00 | -0.02 | -0.24 |
| G155 | GEN | 45.56 | -2.28 | -6.03 |  | G147 | GEN | 1.04 | -0.02 | 0.07 |
| G132 | GEN | 45.56 | -2.23 | -10.59 |  | G84 | GEN | 1.04 | -0.02 | 0.11 |
| G6 | GEN | 45.67 | -2.21 | -3.23 |  | G44 | GEN | 1.03 | -0.02 | 0.06 |
| G2 | GEN | 45.67 | -2.16 | -9.33 |  | G74 | GEN | 1.03 | -0.01 | -0.07 |
| G51 | GEN | 45.78 | -1.73 | -7.61 |  | G100 | GEN | 1.03 | -0.01 | 0.03 |
| G58 | GEN | 46.11 | -1.59 | -3.43 |  | G126 | GEN | 1.03 | -0.01 | 0.12 |
| G107 | GEN | 46.56 | -0.80 | 3.34 |  | G38 | GEN | 1.02 | 0.00 | -0.15 |
| G142 | GEN | 46.56 | -0.70 | -10.15 |  | G39 | GEN | 1.02 | 0.00 | 0.16 |
| G133 | GEN | 46.56 | -0.39 | -10.22 |  | G30 | GEN | 1.03 | 0.00 | -0.17 |
| G86 | GEN | 47.00 | -0.38 | 20.44 |  | G123 | GEN | 1.02 | 0.00 | -0.01 |
| G128 | GEN | 46.89 | -0.02 | -5.17 |  | G113 | GEN | 1.03 | 0.00 | 0.13 |
| G39 | GEN | 47.00 | 0.00 | -5.52 |  | G119 | GEN | 1.01 | 0.01 | 0.02 |
| G38 | GEN | 46.89 | 0.02 | -7.05 |  | G10 | GEN | 1.01 | 0.02 | -0.13 |
| G152 | GEN | 47.00 | 0.07 | -2.44 |  | G46 | GEN | 1.01 | 0.02 | 0.13 |
| G29 | GEN | 47.33 | 0.19 | 8.18 |  | G91 | GEN | 1.01 | 0.02 | -0.08 |
| G37 | GEN | 47.33 | 0.55 | -3.90 |  | G133 | GEN | 1.03 | 0.02 | 0.12 |
| G100 | GEN | 47.22 | 0.76 | -2.47 |  | G22 | GEN | 1.03 | 0.02 | -0.05 |
| G55 | GEN | 47.67 | 0.99 | -4.35 |  | G132 | GEN | 1.02 | 0.02 | 0.11 |
| G153 | GEN | 47.56 | 1.25 | -13.19 |  | G31 | GEN | 1.01 | 0.02 | 0.27 |
| G78 | GEN | 47.89 | 1.35 | 6.95 |  | G148 | GEN | 1.01 | 0.03 | 0.15 |
| G10 | GEN | 47.89 | 1.39 | 5.94 |  | G137 | GEN | 1.01 | 0.03 | -0.02 |
| G21 | GEN | 47.78 | 1.51 | -0.48 |  | G95 | GEN | 1.02 | 0.04 | -0.09 |
| G120 | GEN | 48.11 | 1.55 | 8.59 |  | G13 | GEN | 1.01 | 0.04 | 0.07 |
| G99 | GEN | 48.00 | 1.69 | 4.31 |  | G158 | GEN | 1.01 | 0.04 | 0.11 |
| G91 | GEN | 47.89 | 1.81 | -7.41 |  | G150 | GEN | 1.02 | 0.04 | 0.00 |
| G73 | GEN | 48.22 | 1.91 | 7.43 |  | G71 | GEN | 1.00 | 0.04 | -0.11 |
| G156 | GEN | 47.78 | 1.91 | -14.24 |  | G120 | GEN | 1.00 | 0.05 | 0.03 |
| G114 | GEN | 48.00 | 2.00 | 0.29 |  | G49 | GEN | 0.97 | 0.05 | 0.03 |
| G79 | GEN | 48.22 | 2.02 | -5.65 |  | G135 | GEN | 1.01 | 0.05 | 0.13 |
| G60 | GEN | 48.22 | 2.35 | 0.18 |  | G7 | GEN | 1.02 | 0.05 | -0.25 |
| G70 | GEN | 48.78 | 2.86 | 3.99 |  | G155 | GEN | 1.02 | 0.05 | 0.01 |
| G113 | GEN | 48.56 | 2.91 | -4.36 |  | G93 | GEN | 0.98 | 0.05 | 0.18 |
| G85 | GEN | 49.00 | 3.19 | 1.74 |  | G130 | GEN | 1.00 | 0.05 | -0.05 |
| G74 | GEN | 49.00 | 3.20 | 8.52 |  | G37 | GEN | 1.01 | 0.05 | 0.05 |
| G124 | GEN | 48.89 | 3.55 | -5.42 |  | G66 | GEN | 0.98 | 0.05 | -0.16 |
| G110 | GEN | 49.00 | 3.82 | -7.39 |  | G107 | GEN | 0.98 | 0.06 | -0.02 |
| G22 | GEN | 49.00 | 3.96 | -4.30 |  | G136 | GEN | 1.02 | 0.06 | -0.10 |
| G103 | GEN | 49.33 | 4.19 | 3.36 |  | G24 | GEN | 0.99 | 0.07 | -0.04 |
| G3 | GEN | 49.89 | 4.60 | 8.31 |  | G53 | GEN | 1.00 | 0.07 | 0.10 |
| G19 | GEN | 49.67 | 4.62 | 6.66 |  | G131 | GEN | 0.99 | 0.07 | 0.05 |
| G126 | GEN | 49.44 | 4.63 | -5.52 |  | G146 | GEN | 1.00 | 0.07 | -0.02 |
| G147 | GEN | 49.56 | 4.74 | -4.39 |  | G26 | GEN | 0.99 | 0.08 | 0.02 |
| G40 | GEN | 49.78 | 4.87 | -6.71 |  | G82 | GEN | 0.99 | 0.08 | 0.02 |
| G150 | GEN | 49.89 | 4.90 | -0.48 |  | G20 | GEN | 0.99 | 0.08 | 0.11 |
| G80 | GEN | 49.67 | 5.07 | -0.86 |  | G160 | GEN | 0.98 | 0.08 | -0.16 |
| G84 | GEN | 50.00 | 5.44 | -8.55 |  | G128 | GEN | 0.98 | 0.09 | -0.07 |
| G35 | GEN | 50.22 | 5.56 | -0.94 |  | G127 | GEN | 0.96 | 0.09 | 0.30 |
| G105 | GEN | 50.22 | 5.70 | 0.40 |  | G152 | GEN | 0.99 | 0.09 | -0.05 |
| G146 | GEN | 50.44 | 5.95 | -9.09 |  | G110 | GEN | 0.99 | 0.09 | 0.00 |
| G108 | GEN | 50.44 | 6.04 | 2.51 |  | G19 | GEN | 0.98 | 0.10 | -0.01 |
| G116 | GEN | 50.22 | 6.14 | -11.28 |  | G156 | GEN | 0.99 | 0.10 | 0.09 |
| G9 | GEN | 50.56 | 6.40 | -0.39 |  | G32 | GEN | 0.97 | 0.10 | -0.02 |
| G47 | GEN | 51.00 | 6.52 | 14.10 |  | G35 | GEN | 0.98 | 0.10 | -0.09 |
| G27 | GEN | 50.89 | 6.66 | 6.26 |  | G3 | GEN | 0.93 | 0.11 | -0.09 |
| G119 | GEN | 50.78 | 6.79 | -5.26 |  | G108 | GEN | 0.97 | 0.11 | 0.00 |
| G36 | GEN | 51.22 | 7.17 | 14.40 |  | G42 | GEN | 0.97 | 0.11 | 0.00 |
| G67 | GEN | 51.11 | 7.18 | 1.46 |  | G102 | GEN | 0.99 | 0.11 | -0.02 |
| G31 | GEN | 51.11 | 7.19 | 0.12 |  | G6 | GEN | 0.96 | 0.12 | 0.03 |
| G33 | GEN | 51.33 | 7.22 | 5.59 |  | G145 | GEN | 0.97 | 0.12 | -0.04 |
| G154 | GEN | 51.22 | 7.33 | -0.16 |  | G73 | GEN | 0.94 | 0.12 | -0.14 |
| G145 | GEN | 51.56 | 8.23 | -3.30 |  | G142 | GEN | 0.97 | 0.12 | 0.10 |
| G87 | GEN | 51.78 | 8.53 | -2.60 |  | G149 | GEN | 0.98 | 0.13 | -0.01 |
| G101 | GEN | 51.89 | 8.54 | 7.18 |  | G21 | GEN | 0.96 | 0.14 | -0.06 |
| G64 | GEN | 52.00 | 8.63 | -3.71 |  | G115 | GEN | 0.96 | 0.14 | 0.03 |
| G48 | GEN | 52.11 | 8.76 | 5.26 |  | G69 | GEN | 0.96 | 0.14 | 0.03 |
| G28 | GEN | 51.89 | 8.86 | -8.73 |  | G43 | GEN | 0.93 | 0.14 | 0.14 |
| G17 | GEN | 52.22 | 9.11 | 2.83 |  | G139 | GEN | 0.94 | 0.15 | -0.19 |
| G106 | GEN | 52.33 | 9.16 | 1.94 |  | G114 | GEN | 0.94 | 0.15 | 0.16 |
| G71 | GEN | 52.33 | 9.20 | 5.56 |  | G56 | GEN | 0.94 | 0.16 | 0.13 |
| G7 | GEN | 52.33 | 9.39 | 3.01 |  | G94 | GEN | 0.94 | 0.16 | -0.04 |
| G92 | GEN | 52.22 | 9.87 | -12.87 |  | G138 | GEN | 0.94 | 0.17 | 0.05 |
| G158 | GEN | 52.56 | 10.13 | -20.91 |  | G124 | GEN | 0.93 | 0.17 | -0.08 |
| G13 | GEN | 52.89 | 10.19 | 5.27 |  | G2 | GEN | 0.93 | 0.17 | -0.03 |
| G159 | GEN | 52.78 | 10.25 | -5.38 |  | G129 | GEN | 0.93 | 0.18 | 0.23 |
| G93 | GEN | 53.33 | 10.76 | 3.26 |  | G87 | GEN | 0.92 | 0.18 | 0.10 |
| G52 | GEN | 53.44 | 11.15 | 0.89 |  | G81 | GEN | 0.91 | 0.19 | 0.06 |
| G75 | GEN | 53.56 | 11.73 | 3.48 |  | G143 | GEN | 0.93 | 0.20 | 0.13 |
| G125 | GEN | 54.11 | 12.29 | 4.27 |  | G60 | GEN | 0.94 | 0.21 | 0.15 |
| G50 | GEN | 54.22 | 12.34 | 8.21 |  | G125 | GEN | 0.91 | 0.21 | -0.01 |
| G59 | GEN | 54.22 | 12.60 | 3.92 |  | G28 | GEN | 0.92 | 0.21 | -0.12 |
| G89 | GEN | 54.89 | 13.75 | 1.33 |  | G34 | GEN | 0.89 | 0.22 | -0.24 |
| G1 | GEN | 55.22 | 14.10 | 16.10 |  | G96 | GEN | 0.92 | 0.22 | -0.16 |
| G143 | GEN | 54.89 | 14.11 | -9.21 |  | G5 | GEN | 0.90 | 0.22 | -0.22 |
| G20 | GEN | 55.00 | 14.27 | -4.06 |  | G36 | GEN | 0.91 | 0.23 | -0.03 |
| G26 | GEN | 55.56 | 15.02 | 2.76 |  | G111 | GEN | 0.88 | 0.23 | 0.24 |
| G42 | GEN | 55.78 | 15.43 | 1.59 |  | G55 | GEN | 0.87 | 0.23 | 0.19 |
| G109 | GEN | 56.11 | 15.48 | 6.03 |  | G122 | GEN | 0.88 | 0.24 | -0.08 |
| G68 | GEN | 57.00 | 17.27 | 14.34 |  | G90 | GEN | 0.89 | 0.25 | -0.15 |
| G61 | GEN | 57.22 | 17.63 | 7.59 |  | G70 | GEN | 0.88 | 0.26 | 0.07 |
| G4 | GEN | 57.22 | 18.06 | 1.49 |  | G99 | GEN | 0.86 | 0.28 | -0.10 |
| G134 | GEN | 59.00 | 20.77 | -1.95 |  | G25 | GEN | 0.86 | 0.30 | -0.12 |
| G140 | GEN | 59.44 | 21.89 | -12.02 |  | G8 | GEN | 0.81 | 0.35 | -0.14 |
| G63 | GEN | 60.44 | 23.18 | 7.81 |  | G17 | GEN | 0.81 | 0.37 | -0.24 |
| G62 | GEN | 60.33 | 23.45 | -4.78 |  | G162 | GEN | 0.76 | 0.48 | -0.09 |
| E1 | ENV | 54.26 | 0.58 | -0.66 |  | E1 | ENV | 1.14 | -0.63 | 0.77 |
| E2 | ENV | 42.55 | 0.60 | -0.05 |  | E2 | ENV | 1.00 | -0.38 | -0.18 |
| E3 | ENV | 44.04 | 0.55 | 0.75 |  | E3 | ENV | 0.94 | -0.68 | -0.61 |
