# Supplementary material for: Phenotyping and Genotype × Environment Interaction of Resistance to Leaffolder, Cnaphalocrocis medinalis Guenee (Lepidoptera: Pyralidae) in Rice
Source: Front Plant Sci. 2019 Feb 18;10:49. doi: 10.3389/fpls.2019.00049 (PMC6387916; doi:10.3389/fpls.2019.00049)
Supplement: Supplementary file 4 [file Table_4.DOC]

**Supplementary Table 4. Significant pairwise comparisons of RILs with their parents**

| RESPONSE VARIABLE: DA | | |  |  |  |  | RESPONSE VARIABLE: DS | | |  |  |  |
| --- | --- | --- | --- | --- | --- | --- | --- | --- | --- | --- | --- | --- |
| SIGNIFICANT PAIRWISE COMPARISONS (IF ANY): | | | | |  |  | SIGNIFICANT PAIRWISE COMPARISONS (IF ANY): | | | | |  |
| Compared with control(s) | | |  |  |  |  | Compared with control(s) | | |  |  |  |
|  |  |  |  |  |  |  |  |  |  |  |  |  |
|  | Trmt[i] | Trmt[j] | Difference | Lower | Upper |  |  | Trmt[i] | Trmt[j] | Difference | Lower | Upper |
| 1 | MP10 | TN1 | -345.51 | -462.28 | -228.75 |  | 1 | MP10 | TN1 | -4.67 | -6.62 | -2.71 |
| 2 | MP108 | TN1 | -403.64 | -520.40 | -286.87 |  | 2 | MP108 | TN1 | -5.56 | -7.51 | -3.60 |
| 3 | MP11 | TN1 | -235.79 | -352.55 | -119.03 |  | 3 | MP11 | TN1 | -2.67 | -4.62 | -0.71 |
| 4 | MP110 | TN1 | -214.08 | -330.84 | -97.32 |  | 4 | MP110 | TN1 | -2.00 | -3.95 | -0.05 |
| 5 | MP111 | TN1 | -207.10 | -323.86 | -90.34 |  | 5 | MP111 | TN1 | -2.44 | -4.40 | -0.49 |
| 6 | MP112 | TN1 | -285.35 | -402.11 | -168.59 |  | 6 | MP112 | TN1 | -3.33 | -5.29 | -1.38 |
| 7 | MP114 | TN1 | -438.28 | -555.05 | -321.52 |  | 7 | MP114 | TN1 | -6.00 | -7.95 | -4.05 |
| 8 | MP115 | TN1 | -274.47 | -391.23 | -157.70 |  | 8 | MP115 | TN1 | -3.33 | -5.29 | -1.38 |
| 9 | MP116 | TN1 | -212.80 | -329.56 | -96.03 |  | 9 | MP12 | TN1 | -4.89 | -6.84 | -2.93 |
| 10 | MP117 | TN1 | -131.57 | -248.34 | -14.81 |  | 10 | MP120 | TN1 | -5.33 | -7.29 | -3.38 |
| 11 | MP12 | TN1 | -391.92 | -508.68 | -275.15 |  | 11 | MP122 | TN1 | -4.00 | -5.95 | -2.05 |
| 12 | MP120 | TN1 | -384.08 | -500.85 | -267.32 |  | 12 | MP123 | TN1 | -3.78 | -5.73 | -1.82 |
| 13 | MP121 | TN1 | -142.16 | -258.92 | -25.39 |  | 13 | MP124 | TN1 | -2.00 | -3.95 | -0.05 |
| 14 | MP122 | TN1 | -342.01 | -458.77 | -225.24 |  | 14 | MP126 | TN1 | -3.33 | -5.29 | -1.38 |
| 15 | MP123 | TN1 | -293.68 | -410.44 | -176.91 |  | 15 | MP127 | TN1 | -4.89 | -6.84 | -2.93 |
| 16 | MP124 | TN1 | -192.48 | -309.24 | -75.71 |  | 16 | MP131 | TN1 | -4.44 | -6.40 | -2.49 |
| 17 | MP125 | TN1 | -134.86 | -251.63 | -18.10 |  | 17 | MP133 | TN1 | -2.67 | -4.62 | -0.71 |
| 18 | MP126 | TN1 | -266.63 | -383.40 | -149.87 |  | 18 | MP134 | TN1 | -3.56 | -5.51 | -1.60 |
| 19 | MP127 | TN1 | -376.88 | -493.64 | -260.12 |  | 19 | MP138 | TN1 | -2.89 | -4.84 | -0.93 |
| 20 | MP131 | TN1 | -354.66 | -471.42 | -237.90 |  | 20 | MP14 | TN1 | -3.11 | -5.07 | -1.16 |
| 21 | MP132 | TN1 | -190.99 | -307.75 | -74.22 |  | 21 | MP142 | TN1 | -3.33 | -5.29 | -1.38 |
| 22 | MP133 | TN1 | -248.87 | -365.63 | -132.11 |  | 22 | MP143 | TN1 | -2.44 | -4.40 | -0.49 |
| 23 | MP134 | TN1 | -297.22 | -413.99 | -180.46 |  | 23 | MP144 | TN1 | -3.11 | -5.07 | -1.16 |
| 24 | MP136 | TN1 | -118.31 | -235.07 | -1.54 |  | 24 | MP146 | TN1 | -4.44 | -6.40 | -2.49 |
| 25 | MP138 | TN1 | -245.02 | -361.79 | -128.26 |  | 25 | MP149 | TN1 | -2.22 | -4.18 | -0.27 |
| 26 | MP139 | TN1 | -156.72 | -273.49 | -39.96 |  | 26 | MP15 | TN1 | -5.56 | -7.51 | -3.60 |
| 27 | MP14 | TN1 | -281.05 | -397.82 | -164.29 |  | 27 | MP16 | TN1 | -3.11 | -5.07 | -1.16 |
| 28 | MP142 | TN1 | -302.44 | -419.20 | -185.68 |  | 28 | MP17 | TN1 | -3.33 | -5.29 | -1.38 |
| 29 | MP143 | TN1 | -223.04 | -339.80 | -106.27 |  | 29 | MP18 | TN1 | -3.33 | -5.29 | -1.38 |
| 30 | MP144 | TN1 | -269.20 | -385.96 | -152.43 |  | 30 | MP2 | TN1 | -4.22 | -6.18 | -2.27 |
| 31 | MP145 | TN1 | 172.09 | 55.33 | 288.85 |  | 31 | MP20 | TN1 | -4.89 | -6.84 | -2.93 |
| 32 | MP146 | TN1 | -348.16 | -464.92 | -231.39 |  | 32 | MP206 | TN1 | -2.44 | -4.40 | -0.49 |
| 33 | MP149 | TN1 | -225.62 | -342.38 | -108.86 |  | 33 | MP209 | TN1 | -2.00 | -3.95 | -0.05 |
| 34 | MP15 | TN1 | -425.49 | -542.25 | -308.72 |  | 34 | MP21 | TN1 | -2.00 | -3.95 | -0.05 |
| 35 | MP16 | TN1 | -284.62 | -401.39 | -167.86 |  | 35 | MP211 | TN1 | -3.11 | -5.07 | -1.16 |
| 36 | MP17 | TN1 | -252.07 | -368.83 | -135.30 |  | 36 | MP215 | TN1 | -5.78 | -7.73 | -3.82 |
| 37 | MP18 | TN1 | -282.33 | -399.09 | -165.57 |  | 37 | MP216 | TN1 | -3.11 | -5.07 | -1.16 |
| 38 | MP2 | TN1 | -341.55 | -458.31 | -224.78 |  | 38 | MP217 | TN1 | -5.56 | -7.51 | -3.60 |
| 39 | MP20 | TN1 | -367.24 | -484.01 | -250.48 |  | 39 | MP22 | TN1 | -4.22 | -6.18 | -2.27 |
| 40 | MP206 | TN1 | -229.18 | -345.95 | -112.42 |  | 40 | MP27 | TN1 | -5.33 | -7.29 | -3.38 |
| 41 | MP209 | TN1 | -218.24 | -335.01 | -101.48 |  | 41 | MP32 | TN1 | -3.33 | -5.29 | -1.38 |
| 42 | MP21 | TN1 | -212.01 | -328.78 | -95.25 |  | 42 | MP4 | TN1 | -2.22 | -4.18 | -0.27 |
| 43 | MP211 | TN1 | -266.63 | -383.40 | -149.87 |  | 43 | MP7 | TN1 | -2.44 | -4.40 | -0.49 |
| 44 | MP215 | TN1 | -407.60 | -524.36 | -290.84 |  | 44 | MP8 | TN1 | -2.67 | -4.62 | -0.71 |
| 45 | MP216 | TN1 | -280.25 | -397.01 | -163.48 |  | 45 | MP9 | TN1 | -2.00 | -3.95 | -0.05 |
| 46 | MP217 | TN1 | -383.04 | -499.80 | -266.27 |  | 46 | MP220 | TN1 | -3.11 | -5.07 | -1.16 |
| 47 | MP22 | TN1 | -336.17 | -452.94 | -219.41 |  | 47 | MP221 | TN1 | -2.44 | -4.40 | -0.49 |
| 48 | MP27 | TN1 | -386.61 | -503.37 | -269.85 |  | 48 | MP222 | TN1 | -4.89 | -6.84 | -2.93 |
| 49 | MP31 | TN1 | -124.04 | -240.80 | -7.27 |  | 49 | MP223 | TN1 | -2.89 | -4.84 | -0.93 |
| 50 | MP32 | TN1 | -297.86 | -414.63 | -181.10 |  | 50 | MP224 | TN1 | -3.11 | -5.07 | -1.16 |
| 51 | MP37 | TN1 | 179.20 | 62.44 | 295.96 |  | 51 | MP226 | TN1 | -2.44 | -4.40 | -0.49 |
| 52 | MP4 | TN1 | -215.14 | -331.90 | -98.38 |  | 52 | MP227 | TN1 | -4.00 | -5.95 | -2.05 |
| 53 | MP42 | TN1 | -181.51 | -298.28 | -64.75 |  | 53 | MP230 | TN1 | -3.11 | -5.07 | -1.16 |
| 54 | MP44 | TN1 | -153.95 | -270.71 | -37.18 |  | 54 | MP231 | TN1 | -3.78 | -5.73 | -1.82 |
| 55 | MP46 | TN1 | -123.80 | -240.56 | -7.03 |  | 55 | MP232 | TN1 | -3.78 | -5.73 | -1.82 |
| 56 | MP7 | TN1 | -229.90 | -346.66 | -113.13 |  | 56 | MP235 | TN1 | -2.89 | -4.84 | -0.93 |
| 57 | MP8 | TN1 | -228.94 | -345.70 | -112.17 |  | 57 | MP236 | TN1 | -2.22 | -4.18 | -0.27 |
| 58 | MP9 | TN1 | -191.19 | -307.96 | -74.43 |  | 58 | MP243 | TN1 | -2.00 | -3.95 | -0.05 |
| 59 | MP220 | TN1 | -287.38 | -404.14 | -170.62 |  | 59 | MP244 | TN1 | -3.33 | -5.29 | -1.38 |
| 60 | MP221 | TN1 | -224.03 | -340.80 | -107.27 |  | 60 | MP246 | TN1 | -3.33 | -5.29 | -1.38 |
| 61 | MP222 | TN1 | -366.41 | -483.17 | -249.64 |  | 61 | MP249 | TN1 | -2.22 | -4.18 | -0.27 |
| 62 | MP223 | TN1 | -274.17 | -390.93 | -157.41 |  | 62 | MP300 | TN1 | -3.11 | -5.07 | -1.16 |
| 63 | MP224 | TN1 | -301.73 | -418.50 | -184.97 |  | 63 | MP303 | TN1 | -4.00 | -5.95 | -2.05 |
| 64 | MP226 | TN1 | -229.07 | -345.84 | -112.31 |  | 64 | MP307 | TN1 | -3.33 | -5.29 | -1.38 |
| 65 | MP227 | TN1 | -318.49 | -435.25 | -201.73 |  | 65 | MP313 | TN1 | -2.67 | -4.62 | -0.71 |
| 66 | MP228 | TN1 | -204.89 | -321.65 | -88.13 |  | 66 | MP316 | TN1 | -3.33 | -5.29 | -1.38 |
| 67 | MP230 | TN1 | -284.53 | -401.30 | -167.77 |  | 67 | MP318 | TN1 | -4.00 | -5.95 | -2.05 |
| 68 | MP231 | TN1 | -319.35 | -436.11 | -202.58 |  | 68 | MP320 | TN1 | -3.11 | -5.07 | -1.16 |
| 69 | MP232 | TN1 | -310.26 | -427.02 | -193.49 |  | 69 | MP323 | TN1 | -3.11 | -5.07 | -1.16 |
| 70 | MP233 | TN1 | -213.96 | -330.72 | -97.19 |  | 70 | MP324 | TN1 | -3.56 | -5.51 | -1.60 |
| 71 | MP235 | TN1 | -270.94 | -387.70 | -154.18 |  | 71 | MP325 | TN1 | -5.33 | -7.29 | -3.38 |
| 72 | MP236 | TN1 | -219.63 | -336.40 | -102.87 |  | 72 | MP327 | TN1 | -4.22 | -6.18 | -2.27 |
| 73 | MP237 | TN1 | -128.33 | -245.09 | -11.56 |  | 73 | MP340 | TN1 | -2.00 | -3.95 | -0.05 |
| 74 | MP243 | TN1 | -228.89 | -345.66 | -112.13 |  | 74 | MP352 | TN1 | -2.00 | -3.95 | -0.05 |
| 75 | MP244 | TN1 | -303.30 | -420.06 | -186.54 |  | 75 | MP355 | TN1 | -2.22 | -4.18 | -0.27 |
| 76 | MP246 | TN1 | -286.76 | -403.52 | -170.00 |  | 76 | MP425 | TN1 | -3.11 | -5.07 | -1.16 |
| 77 | MP247 | TN1 | -186.57 | -303.34 | -69.81 |  | 77 | MP435 | TN1 | -2.44 | -4.40 | -0.49 |
| 78 | MP249 | TN1 | -220.69 | -337.45 | -103.93 |  | 78 | MP436 | TN1 | -2.67 | -4.62 | -0.71 |
| 79 | MP300 | TN1 | -271.87 | -388.63 | -155.10 |  | 79 | MP439 | TN1 | -2.00 | -3.95 | -0.05 |
| 80 | MP301 | TN1 | -123.45 | -240.21 | -6.68 |  | 80 | MP444 | TN1 | -3.11 | -5.07 | -1.16 |
| 81 | MP303 | TN1 | -345.03 | -461.79 | -228.26 |  | 81 | MP445 | TN1 | -3.56 | -5.51 | -1.60 |
| 82 | MP307 | TN1 | -285.23 | -401.99 | -168.46 |  | 82 | MP453 | TN1 | -2.22 | -4.18 | -0.27 |
| 83 | MP313 | TN1 | -252.96 | -369.72 | -136.19 |  | 83 | MP457 | TN1 | -4.22 | -6.18 | -2.27 |
| 84 | MP316 | TN1 | -303.24 | -420.01 | -186.48 |  | 84 | MP459 | TN1 | -4.22 | -6.18 | -2.27 |
| 85 | MP317 | TN1 | -144.49 | -261.25 | -27.73 |  | 85 | MP460 | TN1 | -4.00 | -5.95 | -2.05 |
| 86 | MP318 | TN1 | -304.23 | -421.00 | -187.47 |  | 86 | MP520 | TN1 | -2.00 | -3.95 | -0.05 |
| 87 | MP320 | TN1 | -286.46 | -403.23 | -169.70 |  | 87 | MP537 | TN1 | -4.44 | -6.40 | -2.49 |
| 88 | MP323 | TN1 | -275.42 | -392.18 | -158.66 |  | 88 | MP540 | TN1 | -4.89 | -6.84 | -2.93 |
| 89 | MP324 | TN1 | -296.79 | -413.55 | -180.03 |  | 89 | MP544 | TN1 | -4.67 | -6.62 | -2.71 |
| 90 | MP325 | TN1 | -399.71 | -516.47 | -282.94 |  | 90 | MP547 | TN1 | -5.56 | -7.51 | -3.60 |
| 91 | MP327 | TN1 | -359.20 | -475.96 | -242.44 |  | 91 | MP556 | TN1 | -3.11 | -5.07 | -1.16 |
| 92 | MP331 | TN1 | -167.94 | -284.70 | -51.18 |  | 92 | MP558 | TN1 | -3.11 | -5.07 | -1.16 |
| 93 | MP337 | TN1 | -185.25 | -302.01 | -68.49 |  | 93 | W1263 | TN1 | -5.78 | -7.73 | -3.82 |
| 94 | MP338 | TN1 | -190.26 | -307.02 | -73.49 |  | 94 | MP107 | W1263 | 5.78 | 3.83 | 7.73 |
| 95 | MP339 | TN1 | -185.59 | -302.35 | -68.82 |  | 95 | MP11 | W1263 | 3.11 | 1.16 | 5.06 |
| 96 | MP340 | TN1 | -229.16 | -345.92 | -112.40 |  | 96 | MP110 | W1263 | 3.78 | 1.83 | 5.73 |
| 97 | MP342 | TN1 | -187.96 | -304.73 | -71.20 |  | 97 | MP111 | W1263 | 3.33 | 1.38 | 5.29 |
| 98 | MP344 | TN1 | -198.22 | -314.98 | -81.45 |  | 98 | MP112 | W1263 | 2.44 | 0.49 | 4.40 |
| 99 | MP348 | TN1 | -215.95 | -332.71 | -99.18 |  | 99 | MP115 | W1263 | 2.44 | 0.49 | 4.40 |
| 100 | MP352 | TN1 | -197.08 | -313.84 | -80.32 |  | 100 | MP116 | W1263 | 4.00 | 2.05 | 5.95 |
| 101 | MP353 | TN1 | -153.14 | -269.90 | -36.37 |  | 101 | MP117 | W1263 | 4.89 | 2.94 | 6.84 |
| 102 | MP355 | TN1 | -242.42 | -359.18 | -125.66 |  | 102 | MP121 | W1263 | 4.67 | 2.71 | 6.62 |
| 103 | MP361 | TN1 | -231.08 | -347.85 | -114.32 |  | 103 | MP123 | W1263 | 2.00 | 0.05 | 3.95 |
| 104 | MP425 | TN1 | -275.94 | -392.71 | -159.18 |  | 104 | MP124 | W1263 | 3.78 | 1.83 | 5.73 |
| 105 | MP434 | TN1 | 151.30 | 34.53 | 268.06 |  | 105 | MP125 | W1263 | 4.67 | 2.71 | 6.62 |
| 106 | MP435 | TN1 | -236.37 | -353.14 | -119.61 |  | 106 | MP126 | W1263 | 2.44 | 0.49 | 4.40 |
| 107 | MP436 | TN1 | -301.44 | -418.20 | -184.68 |  | 107 | MP132 | W1263 | 4.00 | 2.05 | 5.95 |
| 108 | MP439 | TN1 | -203.30 | -320.06 | -86.53 |  | 108 | MP133 | W1263 | 3.11 | 1.16 | 5.06 |
| 109 | MP440 | TN1 | -190.32 | -307.08 | -73.56 |  | 109 | MP134 | W1263 | 2.22 | 0.27 | 4.17 |
| 110 | MP442 | TN1 | 162.32 | 45.56 | 279.08 |  | 110 | MP135 | W1263 | 5.78 | 3.83 | 7.73 |
| 111 | MP443 | TN1 | -165.04 | -281.80 | -48.28 |  | 111 | MP136 | W1263 | 4.67 | 2.71 | 6.62 |
| 112 | MP444 | TN1 | -265.54 | -382.30 | -148.78 |  | 112 | MP138 | W1263 | 2.89 | 0.94 | 4.84 |
| 113 | MP445 | TN1 | -298.08 | -414.85 | -181.32 |  | 113 | MP139 | W1263 | 4.44 | 2.49 | 6.40 |
| 114 | MP451 | TN1 | -142.89 | -259.65 | -26.13 |  | 114 | MP14 | W1263 | 2.67 | 0.71 | 4.62 |
| 115 | MP453 | TN1 | -219.36 | -336.13 | -102.60 |  | 115 | MP142 | W1263 | 2.44 | 0.49 | 4.40 |
| 116 | MP457 | TN1 | -349.55 | -466.31 | -232.79 |  | 116 | MP143 | W1263 | 3.33 | 1.38 | 5.29 |
| 117 | MP459 | TN1 | -356.66 | -473.42 | -239.90 |  | 117 | MP144 | W1263 | 2.67 | 0.71 | 4.62 |
| 118 | MP460 | TN1 | -327.16 | -443.92 | -210.40 |  | 118 | MP145 | W1263 | 5.78 | 3.83 | 7.73 |
| 119 | MP520 | TN1 | -206.43 | -323.19 | -89.66 |  | 119 | MP148 | W1263 | 4.89 | 2.94 | 6.84 |
| 120 | MP524 | TN1 | -185.56 | -302.33 | -68.80 |  | 120 | MP149 | W1263 | 3.56 | 1.60 | 5.51 |
| 121 | MP528 | TN1 | 230.05 | 113.28 | 346.81 |  | 121 | MP16 | W1263 | 2.67 | 0.71 | 4.62 |
| 122 | MP530 | TN1 | -177.97 | -294.74 | -61.21 |  | 122 | MP17 | W1263 | 2.44 | 0.49 | 4.40 |
| 123 | MP531 | TN1 | -195.45 | -312.21 | -78.69 |  | 123 | MP18 | W1263 | 2.44 | 0.49 | 4.40 |
| 124 | MP533 | TN1 | 139.44 | 22.68 | 256.21 |  | 124 | MP19 | W1263 | 4.89 | 2.94 | 6.84 |
| 125 | MP537 | TN1 | -342.74 | -459.50 | -225.98 |  | 125 | MP206 | W1263 | 3.33 | 1.38 | 5.29 |
| 126 | MP539 | TN1 | -170.65 | -287.41 | -53.89 |  | 126 | MP209 | W1263 | 3.78 | 1.83 | 5.73 |
| 127 | MP540 | TN1 | -374.30 | -491.06 | -257.54 |  | 127 | MP21 | W1263 | 3.78 | 1.83 | 5.73 |
| 128 | MP542 | TN1 | -175.41 | -292.17 | -58.65 |  | 128 | MP211 | W1263 | 2.67 | 0.71 | 4.62 |
| 129 | MP543 | TN1 | -187.97 | -304.73 | -71.21 |  | 129 | MP212 | W1263 | 4.44 | 2.49 | 6.40 |
| 130 | MP544 | TN1 | -343.86 | -460.63 | -227.10 |  | 130 | MP216 | W1263 | 2.67 | 0.71 | 4.62 |
| 131 | MP547 | TN1 | -373.69 | -490.45 | -256.92 |  | 131 | MP23 | W1263 | 5.11 | 3.16 | 7.06 |
| 132 | MP549 | TN1 | -132.98 | -249.75 | -16.22 |  | 132 | MP28 | W1263 | 5.78 | 3.83 | 7.73 |
| 133 | MP555 | TN1 | -226.20 | -342.96 | -109.44 |  | 133 | MP31 | W1263 | 4.89 | 2.94 | 6.84 |
| 134 | MP556 | TN1 | -284.40 | -401.17 | -167.64 |  | 134 | MP32 | W1263 | 2.44 | 0.49 | 4.40 |
| 135 | MP558 | TN1 | -271.31 | -388.07 | -154.54 |  | 135 | MP35 | W1263 | 5.33 | 3.38 | 7.29 |
| 136 | W1263 | TN1 | -380.58 | -497.34 | -263.81 |  | 136 | MP37 | W1263 | 5.78 | 3.83 | 7.73 |
| 137 | MP107 | W1263 | 414.85 | 298.29 | 531.41 |  | 137 | MP4 | W1263 | 3.56 | 1.60 | 5.51 |
| 138 | MP11 | W1263 | 144.79 | 28.23 | 261.35 |  | 138 | MP40 | W1263 | 5.11 | 3.16 | 7.06 |
| 139 | MP110 | W1263 | 166.50 | 49.94 | 283.06 |  | 139 | MP42 | W1263 | 4.00 | 2.05 | 5.95 |
| 140 | MP111 | W1263 | 173.48 | 56.92 | 290.04 |  | 140 | MP44 | W1263 | 4.44 | 2.49 | 6.40 |
| 141 | MP116 | W1263 | 167.78 | 51.22 | 284.34 |  | 141 | MP45 | W1263 | 5.33 | 3.38 | 7.29 |
| 142 | MP117 | W1263 | 249.00 | 132.44 | 365.57 |  | 142 | MP46 | W1263 | 4.22 | 2.27 | 6.17 |
| 143 | MP121 | W1263 | 238.42 | 121.86 | 354.98 |  | 143 | MP7 | W1263 | 3.33 | 1.38 | 5.29 |
| 144 | MP124 | W1263 | 188.10 | 71.54 | 304.66 |  | 144 | MP8 | W1263 | 3.11 | 1.16 | 5.06 |
| 145 | MP125 | W1263 | 245.71 | 129.15 | 362.28 |  | 145 | MP9 | W1263 | 3.78 | 1.83 | 5.73 |
| 146 | MP132 | W1263 | 189.59 | 73.03 | 306.15 |  | 146 | MP220 | W1263 | 2.67 | 0.71 | 4.62 |
| 147 | MP133 | W1263 | 131.71 | 15.14 | 248.27 |  | 147 | MP221 | W1263 | 3.33 | 1.38 | 5.29 |
| 148 | MP135 | W1263 | 392.85 | 276.29 | 509.42 |  | 148 | MP223 | W1263 | 2.89 | 0.94 | 4.84 |
| 149 | MP136 | W1263 | 262.27 | 145.71 | 378.83 |  | 149 | MP224 | W1263 | 2.67 | 0.71 | 4.62 |
| 150 | MP138 | W1263 | 135.55 | 18.99 | 252.12 |  | 150 | MP226 | W1263 | 3.33 | 1.38 | 5.29 |
| 151 | MP139 | W1263 | 223.85 | 107.29 | 340.42 |  | 151 | MP228 | W1263 | 4.22 | 2.27 | 6.17 |
| 152 | MP143 | W1263 | 157.54 | 40.98 | 274.10 |  | 152 | MP230 | W1263 | 2.67 | 0.71 | 4.62 |
| 153 | MP145 | W1263 | 552.67 | 436.10 | 669.23 |  | 153 | MP231 | W1263 | 2.00 | 0.05 | 3.95 |
| 154 | MP148 | W1263 | 308.56 | 192.00 | 425.13 |  | 154 | MP232 | W1263 | 2.00 | 0.05 | 3.95 |
| 155 | MP149 | W1263 | 154.96 | 38.40 | 271.52 |  | 155 | MP233 | W1263 | 4.00 | 2.05 | 5.95 |
| 156 | MP17 | W1263 | 128.51 | 11.95 | 245.07 |  | 156 | MP234 | W1263 | 4.67 | 2.71 | 6.62 |
| 157 | MP19 | W1263 | 286.34 | 169.78 | 402.90 |  | 157 | MP235 | W1263 | 2.89 | 0.94 | 4.84 |
| 158 | MP206 | W1263 | 151.40 | 34.83 | 267.96 |  | 158 | MP236 | W1263 | 3.56 | 1.60 | 5.51 |
| 159 | MP209 | W1263 | 162.33 | 45.77 | 278.90 |  | 159 | MP237 | W1263 | 4.89 | 2.94 | 6.84 |
| 160 | MP21 | W1263 | 168.57 | 52.00 | 285.13 |  | 160 | MP240 | W1263 | 5.78 | 3.83 | 7.73 |
| 161 | MP212 | W1263 | 273.89 | 157.32 | 390.45 |  | 161 | MP241 | W1263 | 5.78 | 3.83 | 7.73 |
| 162 | MP23 | W1263 | 287.87 | 171.31 | 404.43 |  | 162 | MP243 | W1263 | 3.78 | 1.83 | 5.73 |
| 163 | MP28 | W1263 | 354.25 | 237.69 | 470.81 |  | 163 | MP244 | W1263 | 2.44 | 0.49 | 4.40 |
| 164 | MP31 | W1263 | 256.54 | 139.98 | 373.10 |  | 164 | MP245 | W1263 | 5.33 | 3.38 | 7.29 |
| 165 | MP35 | W1263 | 328.04 | 211.48 | 444.60 |  | 165 | MP246 | W1263 | 2.44 | 0.49 | 4.40 |
| 166 | MP37 | W1263 | 559.78 | 443.22 | 676.34 |  | 166 | MP247 | W1263 | 4.44 | 2.49 | 6.40 |
| 167 | MP4 | W1263 | 165.44 | 48.87 | 282.00 |  | 167 | MP248 | W1263 | 4.89 | 2.94 | 6.84 |
| 168 | MP40 | W1263 | 277.62 | 161.05 | 394.18 |  | 168 | MP249 | W1263 | 3.56 | 1.60 | 5.51 |
| 169 | MP42 | W1263 | 199.06 | 82.50 | 315.63 |  | 169 | MP300 | W1263 | 2.67 | 0.71 | 4.62 |
| 170 | MP44 | W1263 | 226.63 | 110.07 | 343.19 |  | 170 | MP301 | W1263 | 4.67 | 2.71 | 6.62 |
| 171 | MP45 | W1263 | 265.27 | 148.71 | 381.83 |  | 171 | MP307 | W1263 | 2.44 | 0.49 | 4.40 |
| 172 | MP46 | W1263 | 256.78 | 140.22 | 373.34 |  | 172 | MP312 | W1263 | 5.78 | 3.83 | 7.73 |
| 173 | MP7 | W1263 | 150.68 | 34.12 | 267.24 |  | 173 | MP313 | W1263 | 3.11 | 1.16 | 5.06 |
| 174 | MP8 | W1263 | 151.64 | 35.08 | 268.20 |  | 174 | MP314 | W1263 | 5.78 | 3.83 | 7.73 |
| 175 | MP9 | W1263 | 189.38 | 72.82 | 305.95 |  | 175 | MP316 | W1263 | 2.44 | 0.49 | 4.40 |
| 176 | MP221 | W1263 | 156.55 | 39.98 | 273.11 |  | 176 | MP317 | W1263 | 4.44 | 2.49 | 6.40 |
| 177 | MP226 | W1263 | 151.50 | 34.94 | 268.07 |  | 177 | MP320 | W1263 | 2.67 | 0.71 | 4.62 |
| 178 | MP228 | W1263 | 175.69 | 59.13 | 292.25 |  | 178 | MP323 | W1263 | 2.67 | 0.71 | 4.62 |
| 179 | MP233 | W1263 | 166.62 | 50.06 | 283.18 |  | 179 | MP324 | W1263 | 2.22 | 0.27 | 4.17 |
| 180 | MP234 | W1263 | 283.40 | 166.84 | 399.96 |  | 180 | MP331 | W1263 | 4.44 | 2.49 | 6.40 |
| 181 | MP236 | W1263 | 160.94 | 44.38 | 277.51 |  | 181 | MP337 | W1263 | 4.44 | 2.49 | 6.40 |
| 182 | MP237 | W1263 | 252.25 | 135.69 | 368.81 |  | 182 | MP338 | W1263 | 4.44 | 2.49 | 6.40 |
| 183 | MP240 | W1263 | 358.20 | 241.63 | 474.76 |  | 183 | MP339 | W1263 | 4.44 | 2.49 | 6.40 |
| 184 | MP241 | W1263 | 393.36 | 276.80 | 509.93 |  | 184 | MP340 | W1263 | 3.78 | 1.83 | 5.73 |
| 185 | MP243 | W1263 | 151.69 | 35.12 | 268.25 |  | 185 | MP342 | W1263 | 4.44 | 2.49 | 6.40 |
| 186 | MP245 | W1263 | 301.70 | 185.14 | 418.26 |  | 186 | MP344 | W1263 | 4.00 | 2.05 | 5.95 |
| 187 | MP247 | W1263 | 194.00 | 77.44 | 310.57 |  | 187 | MP348 | W1263 | 4.00 | 2.05 | 5.95 |
| 188 | MP248 | W1263 | 269.18 | 152.62 | 385.74 |  | 188 | MP352 | W1263 | 3.78 | 1.83 | 5.73 |
| 189 | MP249 | W1263 | 159.89 | 43.32 | 276.45 |  | 189 | MP353 | W1263 | 4.67 | 2.71 | 6.62 |
| 190 | MP301 | W1263 | 257.13 | 140.57 | 373.69 |  | 190 | MP355 | W1263 | 3.56 | 1.60 | 5.51 |
| 191 | MP312 | W1263 | 474.53 | 357.97 | 591.10 |  | 191 | MP357 | W1263 | 5.78 | 3.83 | 7.73 |
| 192 | MP313 | W1263 | 127.62 | 11.06 | 244.18 |  | 192 | MP361 | W1263 | 4.00 | 2.05 | 5.95 |
| 193 | MP314 | W1263 | 469.99 | 353.43 | 586.55 |  | 193 | MP425 | W1263 | 2.67 | 0.71 | 4.62 |
| 194 | MP317 | W1263 | 236.09 | 119.52 | 352.65 |  | 194 | MP434 | W1263 | 5.78 | 3.83 | 7.73 |
| 195 | MP331 | W1263 | 212.64 | 96.08 | 329.20 |  | 195 | MP435 | W1263 | 3.33 | 1.38 | 5.29 |
| 196 | MP337 | W1263 | 195.33 | 78.77 | 311.89 |  | 196 | MP436 | W1263 | 3.11 | 1.16 | 5.06 |
| 197 | MP338 | W1263 | 190.32 | 73.76 | 306.88 |  | 197 | MP439 | W1263 | 3.78 | 1.83 | 5.73 |
| 198 | MP339 | W1263 | 194.99 | 78.43 | 311.55 |  | 198 | MP440 | W1263 | 4.22 | 2.27 | 6.17 |
| 199 | MP340 | W1263 | 151.42 | 34.86 | 267.98 |  | 199 | MP442 | W1263 | 5.78 | 3.83 | 7.73 |
| 200 | MP342 | W1263 | 192.62 | 76.05 | 309.18 |  | 200 | MP443 | W1263 | 4.44 | 2.49 | 6.40 |
| 201 | MP344 | W1263 | 182.36 | 65.80 | 298.92 |  | 201 | MP444 | W1263 | 2.67 | 0.71 | 4.62 |
| 202 | MP348 | W1263 | 164.63 | 48.07 | 281.19 |  | 202 | MP445 | W1263 | 2.22 | 0.27 | 4.17 |
| 203 | MP352 | W1263 | 183.50 | 66.94 | 300.06 |  | 203 | MP448 | W1263 | 5.78 | 3.83 | 7.73 |
| 204 | MP353 | W1263 | 227.44 | 110.88 | 344.00 |  | 204 | MP451 | W1263 | 4.44 | 2.49 | 6.40 |
| 205 | MP355 | W1263 | 138.16 | 21.60 | 254.72 |  | 205 | MP453 | W1263 | 3.56 | 1.60 | 5.51 |
| 206 | MP357 | W1263 | 344.45 | 227.89 | 461.01 |  | 206 | MP520 | W1263 | 3.78 | 1.83 | 5.73 |
| 207 | MP361 | W1263 | 149.50 | 32.93 | 266.06 |  | 207 | MP524 | W1263 | 4.22 | 2.27 | 6.17 |
| 208 | MP434 | W1263 | 531.87 | 415.31 | 648.44 |  | 208 | MP527 | W1263 | 5.78 | 3.83 | 7.73 |
| 209 | MP435 | W1263 | 144.21 | 27.64 | 260.77 |  | 209 | MP528 | W1263 | 5.78 | 3.83 | 7.73 |
| 210 | MP439 | W1263 | 177.28 | 60.72 | 293.84 |  | 210 | MP530 | W1263 | 4.22 | 2.27 | 6.17 |
| 211 | MP440 | W1263 | 190.26 | 73.70 | 306.82 |  | 211 | MP531 | W1263 | 4.22 | 2.27 | 6.17 |
| 212 | MP442 | W1263 | 542.90 | 426.34 | 659.46 |  | 212 | MP533 | W1263 | 5.78 | 3.83 | 7.73 |
| 213 | MP443 | W1263 | 215.54 | 98.97 | 332.10 |  | 213 | MP536 | W1263 | 4.67 | 2.71 | 6.62 |
| 214 | MP448 | W1263 | 338.13 | 221.56 | 454.69 |  | 214 | MP539 | W1263 | 4.44 | 2.49 | 6.40 |
| 215 | MP451 | W1263 | 237.69 | 121.13 | 354.25 |  | 215 | MP541 | W1263 | 5.11 | 3.16 | 7.06 |
| 216 | MP453 | W1263 | 161.21 | 44.65 | 277.78 |  | 216 | MP542 | W1263 | 4.22 | 2.27 | 6.17 |
| 217 | MP520 | W1263 | 174.15 | 57.59 | 290.71 |  | 217 | MP543 | W1263 | 4.00 | 2.05 | 5.95 |
| 218 | MP524 | W1263 | 195.01 | 78.45 | 311.58 |  | 218 | MP546 | W1263 | 5.78 | 3.83 | 7.73 |
| 219 | MP527 | W1263 | 443.33 | 326.76 | 559.89 |  | 219 | MP549 | W1263 | 4.44 | 2.49 | 6.40 |
| 220 | MP528 | W1263 | 610.62 | 494.06 | 727.19 |  | 220 | MP551 | W1263 | 4.89 | 2.94 | 6.84 |
| 221 | MP530 | W1263 | 202.61 | 86.04 | 319.17 |  | 221 | MP553 | W1263 | 5.78 | 3.83 | 7.73 |
| 222 | MP531 | W1263 | 185.13 | 68.56 | 301.69 |  | 222 | MP555 | W1263 | 4.44 | 2.49 | 6.40 |
| 223 | MP533 | W1263 | 520.02 | 403.46 | 636.58 |  | 223 | MP556 | W1263 | 2.67 | 0.71 | 4.62 |
| 224 | MP536 | W1263 | 272.82 | 156.26 | 389.38 |  | 224 | MP558 | W1263 | 2.67 | 0.71 | 4.62 |
| 225 | MP539 | W1263 | 209.93 | 93.36 | 326.49 |  | 225 | TN1 | W1263 | 5.78 | 3.83 | 7.73 |
| 226 | MP541 | W1263 | 327.76 | 211.19 | 444.32 |  |  |  |  |  |  |  |
| 227 | MP542 | W1263 | 205.17 | 88.60 | 321.73 |  | RESPONSE VARIABLE: LL | | |  |  |  |
| 228 | MP543 | W1263 | 192.61 | 76.05 | 309.17 |  | SIGNIFICANT PAIRWISE COMPARISONS (IF ANY): | | | | |  |
| 229 | MP546 | W1263 | 366.20 | 249.64 | 482.77 |  | Compared with control(s) | | |  |  |  |
| 230 | MP549 | W1263 | 247.60 | 131.03 | 364.16 |  |  |  |  |  |  |  |
| 231 | MP551 | W1263 | 265.49 | 148.93 | 382.05 |  |  | Trmt[i] | Trmt[j] | Difference | Lower | Upper |
| 232 | MP553 | W1263 | 497.08 | 380.51 | 613.64 |  | 1 | MP10 | TN1 | 19.96 | 1.97 | 37.96 |
| 233 | MP555 | W1263 | 154.38 | 37.81 | 270.94 |  | 2 | MP11 | TN1 | 21.96 | 3.97 | 39.96 |
| 234 | TN1 | W1263 | 380.58 | 264.02 | 497.14 |  | 3 | MP127 | TN1 | 19.74 | 1.75 | 37.73 |
|  |  |  |  |  |  |  | 4 | MP136 | TN1 | 20.30 | 2.31 | 38.29 |
| RESPONSE VARIABLE: LW | | |  |  |  |  | 5 | MP2 | TN1 | 20.52 | 2.53 | 38.51 |
| SIGNIFICANT PAIRWISE COMPARISONS (IF ANY): | | | | |  |  | 6 | MP216 | TN1 | 18.96 | 0.97 | 36.96 |
| Compared with control(s) | | |  |  |  |  | 7 | MP22 | TN1 | 18.19 | 0.20 | 36.18 |
|  |  |  |  |  |  |  | 8 | MP37 | TN1 | 18.96 | 0.97 | 36.96 |
|  | Trmt[i] | Trmt[j] | Difference | Lower | Upper |  | 9 | MP40 | TN1 | 21.96 | 3.97 | 39.96 |
| 1 | MP107 | TN1 | -0.49 | -0.81 | -0.18 |  | 10 | MP42 | TN1 | 25.08 | 7.08 | 43.07 |
| 2 | MP108 | TN1 | -0.49 | -0.81 | -0.18 |  | 11 | MP44 | TN1 | 25.19 | 7.20 | 43.18 |
| 3 | MP110 | TN1 | -0.53 | -0.84 | -0.21 |  | 12 | MP9 | TN1 | 21.74 | 3.75 | 39.73 |
| 4 | MP111 | TN1 | -0.47 | -0.79 | -0.16 |  | 13 | MP227 | TN1 | 18.30 | 0.31 | 36.29 |
| 5 | MP112 | TN1 | -0.40 | -0.72 | -0.09 |  | 14 | MP245 | TN1 | 19.63 | 1.64 | 37.62 |
| 6 | MP114 | TN1 | -0.62 | -0.93 | -0.30 |  | 15 | MP249 | TN1 | 18.08 | 0.08 | 36.07 |
| 7 | MP115 | TN1 | -0.34 | -0.65 | -0.02 |  | 16 | MP331 | TN1 | 20.85 | 2.86 | 38.84 |
| 8 | MP116 | TN1 | -0.42 | -0.73 | -0.10 |  | 17 | MP436 | TN1 | 18.85 | 0.86 | 36.84 |
| 9 | MP120 | TN1 | -0.42 | -0.73 | -0.10 |  | 18 | MP453 | TN1 | 23.74 | 5.75 | 41.73 |
| 10 | MP122 | TN1 | -0.37 | -0.69 | -0.06 |  | 19 | MP527 | TN1 | 24.19 | 6.20 | 42.18 |
| 11 | MP123 | TN1 | -0.35 | -0.66 | -0.03 |  | 20 | MP531 | TN1 | 19.63 | 1.64 | 37.62 |
| 12 | MP124 | TN1 | -0.62 | -0.93 | -0.30 |  |  |  |  |  |  |  |
| 13 | MP125 | TN1 | -0.43 | -0.74 | -0.11 |  | RESPONSE VARIABLE: LW | | |  |  |  |
| 14 | MP126 | TN1 | -0.45 | -0.76 | -0.13 |  | SIGNIFICANT PAIRWISE COMPARISONS (IF ANY): | | | | | |
| 15 | MP127 | TN1 | -0.44 | -0.75 | -0.12 |  | Compared with control(s) | | |  |  |  |
| 16 | MP131 | TN1 | -0.47 | -0.79 | -0.16 |  | 109 | MP444 | TN1 | -0.43 | -0.74 | -0.11 |
| 17 | MP132 | TN1 | -0.39 | -0.71 | -0.08 |  | 110 | MP445 | TN1 | -0.44 | -0.75 | -0.12 |
| 18 | MP134 | TN1 | -0.44 | -0.75 | -0.12 |  | 111 | MP448 | TN1 | -0.40 | -0.72 | -0.09 |
| 19 | MP135 | TN1 | -0.57 | -0.89 | -0.26 |  | 112 | MP451 | TN1 | -0.39 | -0.71 | -0.08 |
| 20 | MP136 | TN1 | -0.44 | -0.75 | -0.12 |  | 113 | MP453 | TN1 | -0.38 | -0.70 | -0.07 |
| 21 | MP138 | TN1 | -0.32 | -0.63 | 0.00 |  | 114 | MP457 | TN1 | -0.42 | -0.73 | -0.10 |
| 22 | MP139 | TN1 | -0.50 | -0.82 | -0.19 |  | 115 | MP459 | TN1 | -0.40 | -0.72 | -0.09 |
| 23 | MP142 | TN1 | -0.39 | -0.71 | -0.08 |  | 116 | MP460 | TN1 | -0.42 | -0.73 | -0.10 |
| 24 | MP143 | TN1 | -0.42 | -0.73 | -0.10 |  | 117 | MP520 | TN1 | -0.48 | -0.80 | -0.17 |
| 25 | MP144 | TN1 | -0.46 | -0.77 | -0.14 |  | 118 | MP524 | TN1 | -0.48 | -0.80 | -0.17 |
| 26 | MP145 | TN1 | -0.36 | -0.67 | -0.04 |  | 119 | MP527 | TN1 | -0.37 | -0.69 | -0.06 |
| 27 | MP146 | TN1 | -0.54 | -0.85 | -0.22 |  | 120 | MP530 | TN1 | -0.46 | -0.77 | -0.14 |
| 28 | MP148 | TN1 | -0.45 | -0.76 | -0.13 |  | 121 | MP531 | TN1 | -0.49 | -0.81 | -0.18 |
| 29 | MP149 | TN1 | -0.52 | -0.83 | -0.20 |  | 122 | MP536 | TN1 | -0.46 | -0.77 | -0.14 |
| 30 | MP15 | TN1 | -0.42 | -0.73 | -0.10 |  | 123 | MP537 | TN1 | -0.43 | -0.74 | -0.11 |
| 31 | MP16 | TN1 | -0.40 | -0.72 | -0.09 |  | 124 | MP539 | TN1 | -0.38 | -0.70 | -0.07 |
| 32 | MP17 | TN1 | -0.40 | -0.72 | -0.09 |  | 125 | MP540 | TN1 | -0.42 | -0.73 | -0.10 |
| 33 | MP19 | TN1 | -0.39 | -0.71 | -0.08 |  | 126 | MP541 | TN1 | -0.45 | -0.76 | -0.13 |
| 34 | MP2 | TN1 | -0.46 | -0.77 | -0.14 |  | 127 | MP542 | TN1 | -0.40 | -0.72 | -0.09 |
| 35 | MP20 | TN1 | -0.49 | -0.81 | -0.18 |  | 128 | MP544 | TN1 | -0.44 | -0.75 | -0.12 |
| 36 | MP206 | TN1 | -0.39 | -0.71 | -0.08 |  | 129 | MP546 | TN1 | -0.37 | -0.69 | -0.06 |
| 37 | MP209 | TN1 | -0.37 | -0.69 | -0.06 |  | 130 | MP547 | TN1 | -0.35 | -0.66 | -0.03 |
| 38 | MP21 | TN1 | -0.42 | -0.73 | -0.10 |  | 131 | MP549 | TN1 | -0.40 | -0.72 | -0.09 |
| 39 | MP211 | TN1 | -0.36 | -0.67 | -0.04 |  | 132 | MP551 | TN1 | -0.44 | -0.75 | -0.12 |
| 40 | MP215 | TN1 | -0.46 | -0.77 | -0.14 |  | 133 | MP553 | TN1 | -0.33 | -0.64 | -0.01 |
| 41 | MP216 | TN1 | -0.37 | -0.69 | -0.06 |  | 134 | MP555 | TN1 | -0.42 | -0.73 | -0.10 |
| 42 | MP217 | TN1 | -0.40 | -0.72 | -0.09 |  | 135 | MP556 | TN1 | -0.37 | -0.69 | -0.06 |
| 43 | MP22 | TN1 | -0.34 | -0.65 | -0.02 |  | 136 | MP558 | TN1 | -0.45 | -0.76 | -0.13 |
| 44 | MP23 | TN1 | -0.43 | -0.74 | -0.11 |  | 137 | W1263 | TN1 | -0.67 | -0.98 | -0.35 |
| 45 | MP27 | TN1 | -0.33 | -0.64 | -0.01 |  | 138 | MP10 | W1263 | 0.40 | 0.08 | 0.71 |
| 46 | MP28 | TN1 | -0.56 | -0.87 | -0.24 |  | 139 | MP11 | W1263 | 0.38 | 0.07 | 0.70 |
| 47 | MP31 | TN1 | -0.48 | -0.80 | -0.17 |  | 140 | MP115 | W1263 | 0.33 | 0.01 | 0.64 |
| 48 | MP32 | TN1 | -0.36 | -0.67 | -0.04 |  | 141 | MP117 | W1263 | 0.36 | 0.05 | 0.68 |
| 49 | MP35 | TN1 | -0.36 | -0.67 | -0.04 |  | 142 | MP12 | W1263 | 0.38 | 0.07 | 0.70 |
| 50 | MP37 | TN1 | -0.38 | -0.70 | -0.07 |  | 143 | MP121 | W1263 | 0.37 | 0.06 | 0.69 |
| 51 | MP4 | TN1 | -0.48 | -0.80 | -0.17 |  | 144 | MP123 | W1263 | 0.32 | 0.00 | 0.63 |
| 52 | MP42 | TN1 | -0.32 | -0.63 | 0.00 |  | 145 | MP133 | W1263 | 0.40 | 0.08 | 0.71 |
| 53 | MP46 | TN1 | -0.38 | -0.70 | -0.07 |  | 146 | MP138 | W1263 | 0.35 | 0.04 | 0.67 |
| 54 | MP7 | TN1 | -0.45 | -0.76 | -0.13 |  | 147 | MP14 | W1263 | 0.36 | 0.05 | 0.68 |
| 55 | MP220 | TN1 | -0.47 | -0.79 | -0.16 |  | 148 | MP18 | W1263 | 0.36 | 0.05 | 0.68 |
| 56 | MP221 | TN1 | -0.55 | -0.86 | -0.23 |  | 149 | MP212 | W1263 | 0.37 | 0.06 | 0.69 |
| 57 | MP222 | TN1 | -0.43 | -0.74 | -0.11 |  | 150 | MP22 | W1263 | 0.33 | 0.01 | 0.64 |
| 58 | MP224 | TN1 | -0.48 | -0.80 | -0.17 |  | 151 | MP27 | W1263 | 0.34 | 0.02 | 0.66 |
| 59 | MP226 | TN1 | -0.39 | -0.71 | -0.08 |  | 152 | MP40 | W1263 | 0.48 | 0.17 | 0.80 |
| 60 | MP231 | TN1 | -0.32 | -0.63 | 0.00 |  | 153 | MP42 | W1263 | 0.35 | 0.04 | 0.67 |
| 61 | MP233 | TN1 | -0.37 | -0.69 | -0.06 |  | 154 | MP44 | W1263 | 0.47 | 0.16 | 0.79 |
| 62 | MP234 | TN1 | -0.52 | -0.83 | -0.20 |  | 155 | MP45 | W1263 | 0.37 | 0.06 | 0.69 |
| 63 | MP235 | TN1 | -0.44 | -0.75 | -0.12 |  | 156 | MP8 | W1263 | 0.38 | 0.07 | 0.70 |
| 64 | MP236 | TN1 | -0.35 | -0.66 | -0.03 |  | 157 | MP9 | W1263 | 0.46 | 0.15 | 0.78 |
| 65 | MP237 | TN1 | -0.38 | -0.70 | -0.07 |  | 158 | MP223 | W1263 | 0.37 | 0.06 | 0.69 |
| 66 | MP240 | TN1 | -0.35 | -0.66 | -0.03 |  | 159 | MP227 | W1263 | 0.44 | 0.12 | 0.76 |
| 67 | MP241 | TN1 | -0.38 | -0.70 | -0.07 |  | 160 | MP228 | W1263 | 0.50 | 0.18 | 0.81 |
| 68 | MP243 | TN1 | -0.50 | -0.82 | -0.19 |  | 161 | MP230 | W1263 | 0.40 | 0.08 | 0.71 |
| 69 | MP244 | TN1 | -0.35 | -0.66 | -0.03 |  | 162 | MP231 | W1263 | 0.35 | 0.04 | 0.67 |
| 70 | MP245 | TN1 | -0.33 | -0.64 | -0.01 |  | 163 | MP232 | W1263 | 0.42 | 0.10 | 0.73 |
| 71 | MP246 | TN1 | -0.54 | -0.85 | -0.22 |  | 164 | MP236 | W1263 | 0.32 | 0.00 | 0.63 |
| 72 | MP247 | TN1 | -0.42 | -0.73 | -0.10 |  | 165 | MP240 | W1263 | 0.32 | 0.00 | 0.63 |
| 73 | MP248 | TN1 | -0.33 | -0.64 | -0.01 |  | 166 | MP244 | W1263 | 0.32 | 0.00 | 0.63 |
| 74 | MP249 | TN1 | -0.45 | -0.76 | -0.13 |  | 167 | MP245 | W1263 | 0.34 | 0.02 | 0.66 |
| 75 | MP300 | TN1 | -0.48 | -0.80 | -0.17 |  | 168 | MP248 | W1263 | 0.34 | 0.02 | 0.66 |
| 76 | MP301 | TN1 | -0.40 | -0.72 | -0.09 |  | 169 | MP307 | W1263 | 0.34 | 0.02 | 0.66 |
| 77 | MP303 | TN1 | -0.50 | -0.82 | -0.19 |  | 170 | MP312 | W1263 | 0.35 | 0.04 | 0.67 |
| 78 | MP307 | TN1 | -0.33 | -0.64 | -0.01 |  | 171 | MP324 | W1263 | 0.33 | 0.01 | 0.64 |
| 79 | MP312 | TN1 | -0.32 | -0.63 | 0.00 |  | 172 | MP339 | W1263 | 0.36 | 0.05 | 0.68 |
| 80 | MP313 | TN1 | -0.57 | -0.89 | -0.26 |  | 173 | MP348 | W1263 | 0.37 | 0.06 | 0.69 |
| 81 | MP314 | TN1 | -0.39 | -0.71 | -0.08 |  | 174 | MP353 | W1263 | 0.33 | 0.01 | 0.64 |
| 82 | MP316 | TN1 | -0.36 | -0.67 | -0.04 |  | 175 | MP361 | W1263 | 0.34 | 0.02 | 0.66 |
| 83 | MP317 | TN1 | -0.44 | -0.75 | -0.12 |  | 176 | MP528 | W1263 | 0.50 | 0.18 | 0.81 |
| 84 | MP318 | TN1 | -0.38 | -0.70 | -0.07 |  | 177 | MP533 | W1263 | 0.48 | 0.17 | 0.80 |
| 85 | MP320 | TN1 | -0.36 | -0.67 | -0.04 |  | 178 | MP543 | W1263 | 0.46 | 0.15 | 0.78 |
| 86 | MP323 | TN1 | -0.38 | -0.70 | -0.07 |  | 179 | MP547 | W1263 | 0.32 | 0.00 | 0.63 |
| 87 | MP324 | TN1 | -0.34 | -0.65 | -0.02 |  | 180 | MP553 | W1263 | 0.34 | 0.02 | 0.66 |
| 88 | MP325 | TN1 | -0.45 | -0.76 | -0.13 |  | 181 | TN1 | W1263 | 0.67 | 0.35 | 0.98 |
| 89 | MP327 | TN1 | -0.46 | -0.77 | -0.14 |  |  |  |  |  |  |  |
| 90 | MP331 | TN1 | -0.37 | -0.69 | -0.06 |  |  |  |  |  |  |  |
| 91 | MP337 | TN1 | -0.44 | -0.75 | -0.12 |  |  |  |  |  |  |  |
| 92 | MP338 | TN1 | -0.55 | -0.86 | -0.23 |  |  |  |  |  |  |  |
| 93 | MP340 | TN1 | -0.39 | -0.71 | -0.08 |  |  |  |  |  |  |  |
| 94 | MP342 | TN1 | -0.48 | -0.80 | -0.17 |  |  |  |  |  |  |  |
| 95 | MP344 | TN1 | -0.47 | -0.79 | -0.16 |  |  |  |  |  |  |  |
| 96 | MP352 | TN1 | -0.36 | -0.67 | -0.04 |  |  |  |  |  |  |  |
| 97 | MP353 | TN1 | -0.34 | -0.65 | -0.02 |  |  |  |  |  |  |  |
| 98 | MP355 | TN1 | -0.42 | -0.73 | -0.10 |  |  |  |  |  |  |  |
| 99 | MP357 | TN1 | -0.43 | -0.74 | -0.11 |  |  |  |  |  |  |  |
| 100 | MP361 | TN1 | -0.33 | -0.64 | -0.01 |  |  |  |  |  |  |  |
| 101 | MP425 | TN1 | -0.55 | -0.86 | -0.23 |  |  |  |  |  |  |  |
| 102 | MP434 | TN1 | -0.40 | -0.72 | -0.09 |  |  |  |  |  |  |  |
| 103 | MP435 | TN1 | -0.49 | -0.81 | -0.18 |  |  |  |  |  |  |  |
| 104 | MP436 | TN1 | -0.52 | -0.83 | -0.20 |  |  |  |  |  |  |  |
| 105 | MP439 | TN1 | -0.39 | -0.71 | -0.08 |  |  |  |  |  |  |  |
| 106 | MP440 | TN1 | -0.47 | -0.79 | -0.16 |  |  |  |  |  |  |  |
| 107 | MP442 | TN1 | -0.45 | -0.76 | -0.13 |  |  |  |  |  |  |  |
| 108 | MP443 | TN1 | -0.49 | -0.81 | -0.18 |  |  |  |  |  |  |  |
