# Supplementary material for: Phenotyping and Genotype × Environment Interaction of Resistance to Leaffolder, Cnaphalocrocis medinalis Guenee (Lepidoptera: Pyralidae) in Rice
Source: Front Plant Sci. 2019 Feb 18;10:49. doi: 10.3389/fpls.2019.00049 (PMC6387916; doi:10.3389/fpls.2019.00049)
Supplement: Supplementary file 5 [file Table_5.DOC]

**Supplementary Table 5.** ANOVA for AMMI analysis or GGE analysis using multiplicative models

| ------------------------------ | | |  |  |  |  |  |
| --- | --- | --- | --- | --- | --- | --- | --- |
| RESPONSE VARIABLE: DA | | | |  |  |  |  |
| ------------------------------ | | |  |  |  |  |  |
| AMMI ANALYSIS: | | |  |  |  |  |  |
| percent acum Df Sum.Sq Mean.Sq F.value Pr.F | | | | |  |  |  |
| PC1 | 68.1 | 68.1 | 162.00 | 1161193.00 | 7167.86 | 3.08 | 0.00E+00 |
| PC2 | 31.9 | 100 | 160.00 | 544614.00 | 3403.84 | 1.46 | 5.00E-04 |
| PC3 | 0 | 100 | 158.00 | 0.00 | 0.00 | 0 | 1.00E+00 |
|  |  |  |  |  |  |  |  |
| GGE ANALYSIS: | |  |  |  |  |  |  |
|  | percent | acum | Df | Sum.Sq | Mean.Sq | F.value | Pr.F |
| PC1 | 93.9 | 93.9 | 162.00 | 25778901.90 | 159129.02 | 68.31 | 0 |
| PC2 | 4.2 | 98.1 | 160.00 | 1161173.80 | 7257.34 | 3.12 | 0 |
| PC3 | 1.9 | 100 | 158.00 | 527200.30 | 3336.71 | 1.43 | 0.001 |
|  |  |  |  |  |  |  |  |
| ------------------------------ | | |  |  |  |  |  |
| RESPONSE VARIABLE: DS | | | |  |  |  |  |
| ------------------------------ | | |  |  |  |  |  |
| AMMI ANALYSIS: | | |  |  |  |  |  |
| percent acum Df Sum.Sq Mean.Sq F.value Pr.F | | | | |  |  |  |
| PC1 | 57.3 | 57.3 | 162.00 | 274.03 | 1.69 | 2.95 | 0 |
| PC2 | 42.7 | 100 | 160.00 | 204.12 | 1.28 | 2.22 | 0 |
| PC3 | 0 | 100 | 158.00 | 0.00 | 0.00 | 0 | 1 |
| GGE ANALYSIS: | |  |  |  |  |  |  |
| percent acum Df Sum.Sq Mean.Sq F.value Pr.F | | | | |  |  |  |
| PC1 | 88.2 | 88.2 | 162.00 | 3573.37 | 22.06 | 38.46 | 0 |
| PC2 | 6.7 | 94.9 | 160.00 | 273.29 | 1.71 | 2.98 | 0 |
| PC3 | 5 | 99.9 | 158.00 | 203.79 | 1.29 | 2.25 | 0 |
|  |  |  |  |  |  |  |  |
| ------------------------------ | | |  |  |  |  |  |
| RESPONSE VARIABLE: LW | | | |  |  |  |  |
| ------------------------------ | | |  |  |  |  |  |
| AMMI ANALYSIS: | | | |  |  |  |  |
| percent acum Df Sum.Sq Mean.Sq F.value Pr.F | | | | |  |  |  |
| PC1 | 53.3 | 53.3 | 162.00 | 6.61 | 0.04 | 0 | 1 |
| PC2 | 46.7 | 100 | 160.00 | 5.80 | 0.04 | 0 | 1 |
| PC3 | 0 | 100 | 158.00 | 0.00 | 0.00 | 0 | 1 |
| GGE ANALYSIS: | |  |  |  |  |  |  |
| percent acum Df Sum.Sq Mean.Sq F.value Pr.F | | | | |  |  |  |
| PC1 | 52.9 | 52.9 | 162.00 | 13.49 | 0.08 | 0.01 | 1 |
| PC2 | 25.9 | 78.8 | 160.00 | 6.61 | 0.04 | 0 | 1 |
| PC3 | 21.2 | 100 | 158.00 | 5.39 | 0.03 | 0 | 1 |
| ------------------------------ | | |  |  |  |  |  |
| RESPONSE VARIABLE: LL | | | |  |  |  |  |
| ------------------------------ | | |  |  |  |  |  |
| AMMI ANALYSIS: | | |  |  |  |  |  |
|  | percent | acum | Df | Sum.Sq | Mean.Sq | F.value | Pr.F |
| PC1 | 60.4 | 60.4 | 162.00 | 24479.90 | 151.11 | 15578.4 | 0 |
| PC2 | 39.6 | 100 | 160.00 | 16044.21 | 100.28 | 10337.77 | 0 |
| PC3 | 0 | 100 | 158.00 | 0.00 | 0.00 | 0 | 1 |
| GGE ANALYSIS: | |  |  |  |  |  |  |
| percent acum Df Sum.Sq Mean.Sq F.value Pr.F | | | | |  |  |  |
| PC1 | 51.9 | 51.9 | 162.00 | 43669.36 | 269.56 | 27790.1 | 0 |
| PC2 | 29.1 | 81 | 160.00 | 24469.37 | 152.93 | 15766.35 | 0 |
| PC3 | 19 | 100 | 158.00 | 16025.98 | 101.43 | 10456.73 | 0 |
