# Supplementary material for: Phenotyping and Genotype × Environment Interaction of Resistance to Leaffolder, Cnaphalocrocis medinalis Guenee (Lepidoptera: Pyralidae) in Rice
Source: Front Plant Sci. 2019 Feb 18;10:49. doi: 10.3389/fpls.2019.00049 (PMC6387916; doi:10.3389/fpls.2019.00049)
Supplement: Supplementary file 6 [file Table_6.DOC]

| **Suppl. Table 6. Weather parameters during three crop seasons** | | | | | | | | | | | | |
| --- | --- | --- | --- | --- | --- | --- | --- | --- | --- | --- | --- | --- |
| **Season** | **Months** | **Temperature (^O^C)** | | | **R.H. (%)** | | **Rain-Fall**  **(mm)** | **Rainy Days** | **Sun-Shine (Hrs.)** | **Wind Speed**  **(Km/Hr)** | **Evapo-Ration**  **(mm)** | **Crop stage** |
|  |  | **Max.** | **Min.** | **Mean Temp.** | **I** | **II** |  |  |  |  |  |  |
| ***Wet season 2013***  ***(E1)*** | Jul-13 | 32.64 | 23.92 | 28.25 | 81.83 | 56.83 | 150.20 | 9.00 | 4.56 | 10.55 | 4.69 | Sowing |
|  | Aug-13 | 28.49 | 21.94 | 25.21 | 89.90 | 75.93 | 158.10 | 1.00 | 3.39 | 6.36 | 4.02 | Transplanting |
|  | Sep-13 | 31.05 | 20.59 | 25.82 | 87.07 | 64.33 | 110.60 | 8.00 | 5.71 | 3.05 | 4.69 | Vegetative stage – Phenotyping |
|  | Oct-13 | 29.99 | 19.73 | 24.86 | 88.77 | 63.33 | 253.20 | 9.00 | 5.45 | 2.87 | 3.86 | Heading |
|  | Nov-13 | 28.42 | 14.38 | 21.40 | 86.27 | 50.43 | 31.00 | 2.00 | 6.66 | 1.72 | 2.69 | Harvesting |
|  | **Mean** | **30.54** | **21.54** | **26.04** | **86.89** | **65.11** | **168.03** | **6.75** | **4.78** | **5.71** | **4.32** |  |
| ***Dry season 2013***  ***(E2)*** | Dec-13 | 28.02 | 10.09 | 19.05 | 83.10 | 36.58 | 0.00 | 0.00 | 8.87 | 1.75 | 2.70 | Sowing |
|  | Jan-14 | 28.69 | 13.25 | 20.97 | 84.74 | 40.29 | 0.00 | 0.00 | 8.17 | 2.49 | 3.07 | Transplanting |
|  | Feb-14 | 31.20 | 16.55 | 23.88 | 78.36 | 32.75 | 0.00 | 0.00 | 8.99 | 2.93 | 4.62 | Vegetative stage |
|  | Mar-14 | 33.23 | 20.36 | 26.80 | 79.58 | 36.39 | 56.80 | 5.00 | 7.35 | 2.69 | 4.61 | Vegetative stage- Phenotyping |
|  | Apr-14 | 37.60 | 22.04 | 29.82 | 76.73 | 36.07 | 72.60 | 2.00 | 7.72 | 2.02 | 6.02 | Heading |
|  | May-14 | 37.74 | 23.87 | 30.81 | 66.10 | 33.84 | 40.10 | 3.00 | 8.39 | 3.47 | 7.11 | Heading |
|  | Jun-14 | 37.02 | 24.60 | 30.81 | 68.57 | 45.07 | 53.60 | 3.00 | 7.90 | 10.17 | 8.14 | Harvesting |
|  | **Mean** | **33.69** | **19.22** | **26.45** | **77.10** | **35.87** | **44.62** | **2.00** | **8.13** | **2.72** | **5.09** |  |
| ***Wet season 2014***  ***(E3)*** | Jul-14 | 31.80 | 23.60 | 27.70 | 82.32 | 62.40 | 108.00 | 8.00 | 3.20 | 12.40 | 4.00 | Sowing |
|  | Aug-14 | 32.00 | 23.30 | 27.65 | 85.90 | 62.70 | 184.10 | 10.00 | 5.40 | 7.10 | 3.00 | Transplanting |
|  | Sep-14 | 30.30 | 22.40 | 26.35 | 87.43 | 60.80 | 60.60 | 7.00 | 5.10 | 5.20 | 3.00 | Vegetative stage- Phenotyping |
|  | Oct-14 | 31.60 | 19.90 | 25.75 | 83.45 | 49.80 | 69.20 | 3.00 | 6.40 | 2.40 | 4.80 | Heading |
|  | Nov-14 | 30.60 | 16.80 | 23.70 | 79.23 | 40.90 | 10.60 | 1.00 | 7.00 | 1.80 | 4.70 | Harvesting |
|  | **Mean** | **31.26** | **21.20** | **26.23** | **83.67** | **55.32** | **86.50** | **5.80** | **5.42** | **5.78** | **3.90** |  |
