# Supplementary material for: Phenotyping and Genotype × Environment Interaction of Resistance to Leaffolder, Cnaphalocrocis medinalis Guenee (Lepidoptera: Pyralidae) in Rice
Source: Front Plant Sci. 2019 Feb 18;10:49. doi: 10.3389/fpls.2019.00049 (PMC6387916; doi:10.3389/fpls.2019.00049)
Supplement: Supplementary file 7 [file Image_1.pdf]

Supplementary Fig.1 Polygon views of the GGE biplot based on symmetrical scaling for 'which won-where' pattern of rice genotypes in three environments

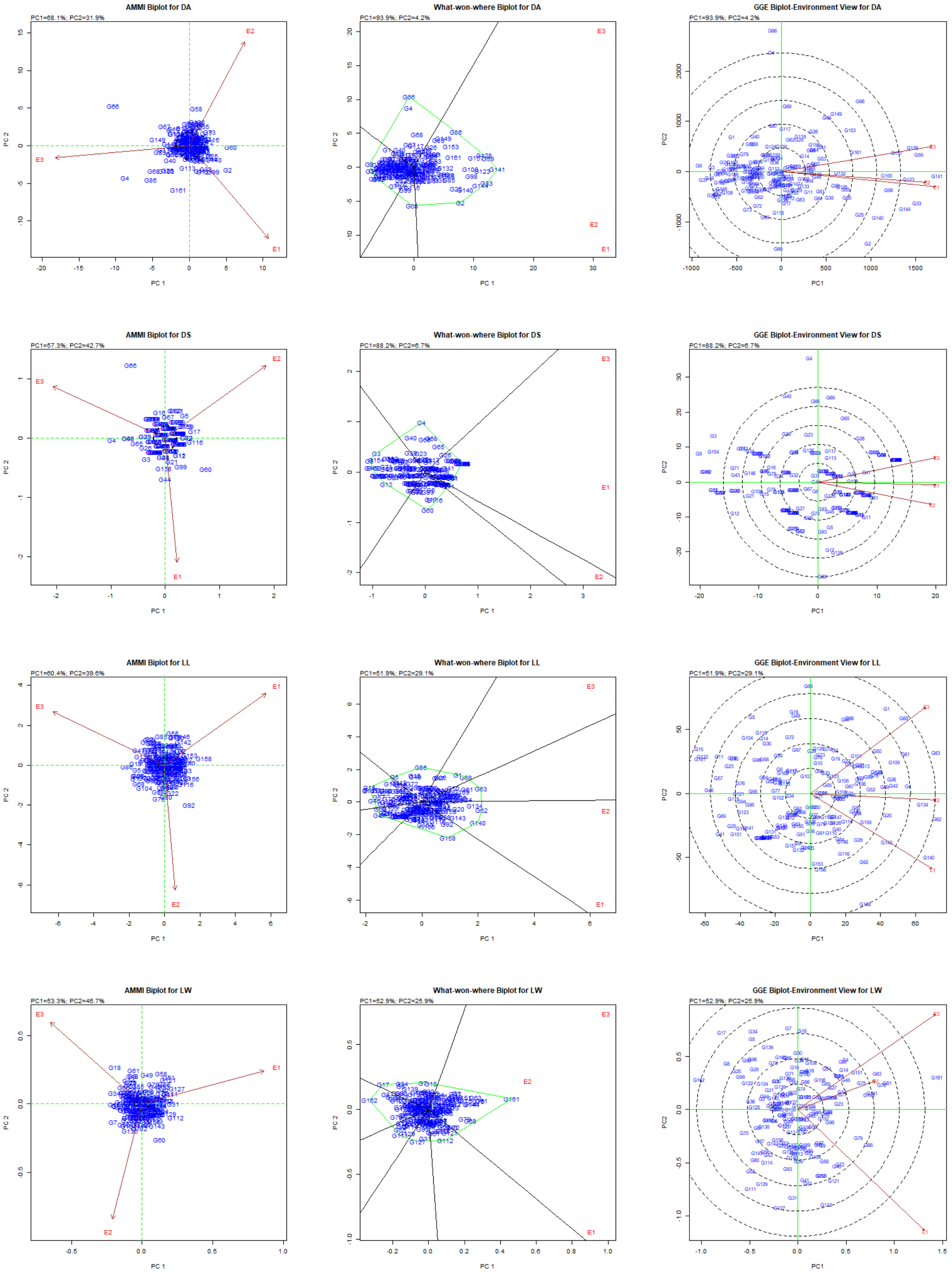

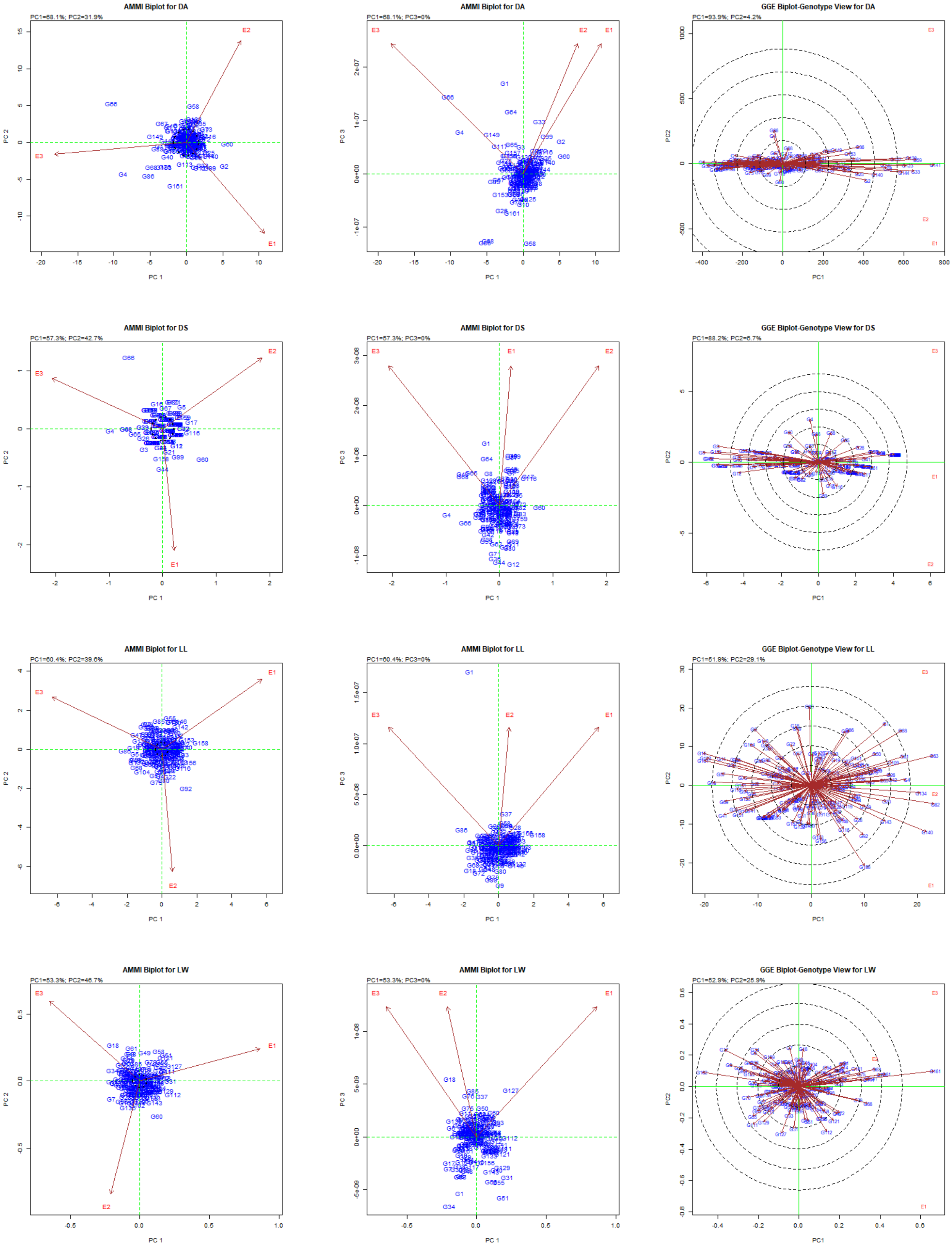

DA = Damaged area; DS = Damage score; LL = Leaf length; LW = Leaf width; E1= Wet season 2013; E2= Dry season 2013-14; E3 = Wet season 2014
